# Concurrent action of purifying selection and gene conversion results in extreme conservation of the major stress-inducible Hsp70 genes in mammals

**DOI:** 10.1038/s41598-018-23508-x

**Published:** 2018-03-23

**Authors:** Kyle Hess, Ryan Oliverio, Peter Nguyen, Dat Le, Jacqueline Ellis, Brianna Kdeiss, Sara Ord, Dimitra Chalkia, Nikolas Nikolaidis

**Affiliations:** 1 Department of Biological Science, Center for Applied Biotechnology Studies, and Center for Computational and Applied Mathematics, California State University, Fullerton, Fullerton, CA 92834 USA; 2 UCLA Center for Systems Biomedicine, Division of Digestive Diseases, School of Medicine, Los Angeles, CA USA; 3 Present Address: Department of Genome Sciences, University of Washington, Seattle, WA USA

## Abstract

Several evolutionary mechanisms alter the fate of mutations and genes within populations based on their exhibited functional effects. To understand the underlying mechanisms involved in the evolution of the cellular stress response, a very conserved mechanism in the course of organismal evolution, we studied the patterns of natural genetic variation and functional consequences of polymorphisms of two stress-inducible Hsp70 genes. These genes, *HSPA1A* and *HSPA1B*, are major orchestrators of the cellular stress response and are associated with several human diseases. Our phylogenetic analyses revealed that the duplication of *HSPA1A* and *HSPA1B* originated in a lineage proceeding to placental mammals, and henceforth they remained in conserved synteny. Additionally, analyses of synonymous and non-synonymous changes suggest that purifying selection shaped the *HSPA1* gene diversification, while gene conversion resulted in high sequence conservation within species. In the human *HSPA1*-cluster, the vast majority of mutations are synonymous and specific genic regions are devoid of mutations. Furthermore, functional characterization of several human polymorphisms revealed subtle differences in HSPA1A stability and intracellular localization. Collectively, the observable patterns of *HSPA1A*-*1B* variation describe an evolutionary pattern, in which purifying selection and gene conversion act simultaneously and conserve a major orchestrator of the cellular stress response.

## Introduction

The cellular stress response (CSR) is a multiprotein system that allows cells and eventually organisms to cope with stress, adapt, and thrive^1,2^. Many of the CSR’s protective functions are orchestrated by a highly-conserved family of proteins known as 70-kDa heat shock proteins (Hsp70s)^1–3^. Hsp70s are molecular chaperones that play crucial roles in cellular function, adaptability, and phenotypic plasticity^4–6^.

A hallmark of Hsp70s is their remarkably high levels of amino acid sequence conservation during organismal evolution^5,7–9^. This feature is usually attributed to the action of purifying selection, most probably due to functional constraints to preserve the primary function of Hsp70s, i.e., refolding of misfolded or stress-denatured proteins.

The evolution of Hsp70s, which has been a subject of intense research, is characterized by several opposing phenomena. For example, the primary function of Hsp70s, protein folding and refolding, is well conserved in all species studied^3,10,11^. Nevertheless, Hsp70s have evolved other secondary functions, i.e., lipid binding, which differentiate even closely related Hsp70s^5,12^. Furthermore, several *hsp70s* show differences in their expression patterns revealing a history of diversifying selection that resulted in several neofunctionalization events^3,8,10,11^. Hsp70s also represent characteristic examples of divergent evolution because differently localized Hsp70s (cytosolic, mitochondria, endoplasmic reticulum) form deep phylogenetic clades dating back to the first eukaryotes^3,8–10,13^. Lastly, studies on the evolution of cytosolic Hsp70s revealed that heat-inducibilty has evolved independently more than once in different species^13,14^, suggesting convergent evolution. Therefore, Hsp70s can be used as a model to study multiple evolutionary mechanisms.

Within the cytosolic Hsp70s, the constitutively expressed and the heat-inducible genes evolve differently. While the former genes are considered as typical examples of evolution by the birth-and-death model, the latter are almost identical in both amino acid and nucleotide sequences^7–9,15^. This feature suggests that the stress-inducible *hsp70* gene copies are evolving in concert under a mechanism known as gene conversion^9,15–17^.

However, closer inspection of the stress-inducible *hsp70* genes in species with three or more copies (e.g., nematodes and flies) revealed that their nucleotide sequences are not identical and several synonymous mutations are present^9,15^. This bias towards silent variation suggests in addition to gene conversion, which homogenizes the *hsp70* sequences, purifying selection shapes diversification by eliminating amino acid altering mutations. Although this property is well supported in nematodes and flies it is less evident in humans and mice, which have only two stress-inducible *hsp70* gene copies, namely *HSPA1A* and *HSPA1B*, organized in tandem. Detailed studies using mouse, rat, and human sequences suggested concerted evolution by the gene conversion mechanism^16^. However, it is not clear whether these genes are also experiencing purifying selection. Furthermore, because the mixed pattern of evolution makes the identification of orthologous sequences difficult, the origin of the *HSPA1A-1B* cluster is yet to be established^8,18^.

In contrast to the well-studied evolution of Hsp70s between species, their evolution and natural variation within a species including humans remains almost completely unknown. A few studies aimed to associate specific *HSPA1A-1B* polymorphisms with particular human diseases^19–21^. However, these studies neither investigated how purifying selection and gene-conversion manifest their action, nor determined whether evolutionary patterns observed between species hold true within species. Furthermore, only a few studies experimentally assessed the impact of mutations on the function/expression of human *HSPA1A-1B*^19–21^. Therefore, how natural mutations manifest their functional consequences and whether they are subject to purifying selection, gene conversion, or both within the human population remains unknown.

To elucidate which genetic and molecular mechanisms govern the evolution of these proteins, we first determined their origin and evolution between species. We then examined whether these same mechanisms can explain the patterns of extant natural variation in humans. Lastly, we assessed the functional consequences of *HSPA1A-1B* natural polymorphisms in humans.

## Results

### Origin and evolution of the *HSPA1A* and *HSPA1B* genes

#### Phylogenetic and syntenic relationships of *HSPA1A*

**Figure 1:**
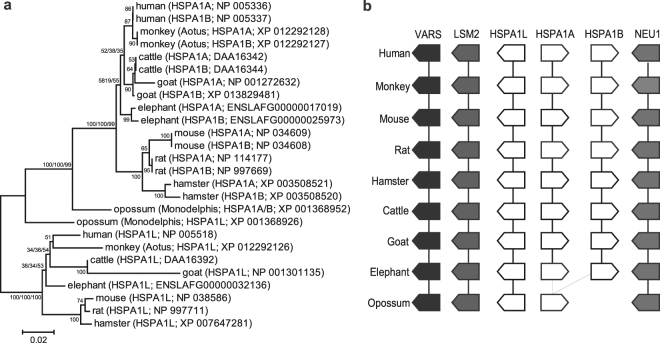
The HspA1A-HspA1B cluster originated early during the evolution of placental mammals and shows intraspecies phylogenetic clades. (**a**) Evolutionary relationships between several mammalian Hsp70s. The evolutionary history was inferred using the Neighbor-Joining (NJ; shown here), Maximum Likelihood (ML), and Maximum Parsimony (MP) methods. The percentage of replicate trees in which the sequences clustered together in the bootstrap test (1000 replicates). Branches supported by all three methods include three bootstrap values (NJ/ML/MP). The evolutionary distances were computed using the JTT matrix-based method and the rate variation among sites was modeled with a gamma distribution (shape parameter = 0.22). All positions containing gaps and missing data were eliminated. (**b**) The HspA1 genes, HspA1A, HspA1B, and HspA1L are in conserved synteny in all mammals. The collected proteins were mapped to the corresponding mammalian genome sequence and the genomic organization of the HspA1 genes was identified and compared to determine syntenic relationships.

To determine the origin and evolution of the human *HSPA1A-1B* gene cluster we used phylogenetic and synteny analyses. The results of these analyses can be summarized as follows. First, the *HspA1A-1B* gene cluster initially appears in placental mammals (Fig. 1). Differently both monotremes and marsupials have only one copy of the gene cluster as well as the neighboring *HspA1L* gene (Fig. 1 and Supplementary Fig. S1). Second, the paralogous *HspA1A-1B* sequences show intraspecies clustering in all placental mammals. In contrast, the *HspA1L* genes show interspecies clustering in which the orthologous sequences form a monophyletic clade (Fig. 1a). And third, the synteny of the *hsp70* genes is conserved in all mammals (Fig. 1b).

#### Nucleotide and amino acid identities of *HSPA1A* and its homologs

The high level of sequence conservation revealed by the phylogenetic analyses can be explained either by strong purifying selection, gene conversion, or a combination of both mechanisms. To distinguish between these possibilities, we used nucleotide and protein analyses. These analyses revealed that in all sequence comparisons the amino acid identity is considerably higher than the nucleotide identity (Table 1 and Supplementary Table S1), a pattern suggestive of purifying selection. To further evaluate this result, we calculated synonymous and non-synonymous distances (ps and pn; Table 2 and Supplementary Table S2). This analysis revealed that in all comparisons the synonymous distances are higher than the non-synonymous, further suggesting purifying selection.

**Table 1:** Sequence comparisons between representative *HspA1* cluster genes reveal that the amino acid identity is considerably higher than the nucleotide identity. Nucleotide and amino acid pairwise identities were calculated with MEGA 6.0. Nucleotide identities are showed above the diagonal and amino acid identities below the diagonal. opos: opossum.

	human 1A	human 1B	human 1L	goat 1A	goat 1B	goat 1L	opos 1	opos 1L
human 1A	—	99.73	81.16	94.14	94.56	78.54	78.54	80.37
human 1B	100.00	—	81.11	94.19	94.61	78.49	78.44	80.22
human 1L	89.95	89.95	—	79.49	80.01	88.64	81.94	80.79
goat 1A	97.65	97.65	88.70	—	98.95	77.55	77.44	79.28
goat 1B	98.74	98.74	89.80	98.74	—	78.02	77.97	79.75
goat 1L	86.50	86.50	92.15	85.40	86.50	—	80.06	79.38
opos 1	94.66	94.66	90.27	92.94	94.20	87.13	—	92.09
opos 1L	92.31	92.31	92.31	91.05	92.15	88.38	93.09	—

**Table 2:** Synonymous (ps; below diagonal) and non-synonymous (pn; above diagonal) distances (and their standard errors) computed using the modified Nei-Gojobori method between representative *HspA1* cluster genes show that the ps values are considerably higher than the pn ones. opos: opossum.

	human 1A	human 1B	human 1L	goat 1A	goat 1B	goat 1L	opos 1	opos 1L
human 1A	—	0.00 ± 0.000	0.07 ± 0.009	0.01 ± 0.004	0.01 ± 0.003	0.09 ± 0.009	0.05 ± 0.008	0.04 ± 0.007
human 1B	0.01 ± 0.004	—	0.06 ± 0.009	0.01 ± 0.004	0.01 ± 0.003	0.09 ± 0.009	0.05 ± 0.008	0.04 ± 0.007
human 1L	0.48 ± 0.021	0.49 ± 0.021	—	0.07 ± 0.009	0.06 ± 0.009	0.04 ± 0.006	0.05 ± 0.007	0.06 ± 0.009
goat 1A	0.16 ± 0.016	0.16 ± 0.016	0.52 ± 0.022	—	0.01 ± 0.002	0.09 ± 0.009	0.06 ± 0.008	0.05 ± 0.006
goat 1B	0.16 ± 0.016	0.16 ± 0.015	0.52 ± 0.022	0.02 ± 0.006	—	0.09 ± 0.009	0.05 ± 0.008	0.04 ± 0.006
goat 1L	0.52 ± 0.022	0.53 ± 0.022	0.28 ± 0.020	0.54 ± 0.023	0.54 ± 0.022	—	0.08 ± 0.007	0.08 ± 0.009
opos 1	0.59 ± 0.023	0.60 ± 0.023	0.49 ± 0.021	0.62 ± 0.023	0.62 ± 0.023	0.50 ± 0.021	—	0.04 ± 0.007
opos 1L	0.56 ± 0.022	0.57 ± 0.022	0.49 ± 0.023	0.58 ± 0.022	0.58 ± 0.023	0.50 ± 0.021	0.16 ± 0.017	—

Nevertheless, purifying selection alone cannot fully explain the intraspecies clades of all paralogous *HspA1A*-*HspA1B* genes. If there were only selection, then to explain the intraspecies phylogenetic clades one would have to assume that the genes are products of very recent gene duplications. However, the presence of the *HspA1A-1B* cluster in all placental mammals, suggests that the gene duplication preceded their speciation events, and thus may be older than 100 million years^22^. Therefore, the most plausible explanation of the phylogenetic incongruence between orthologs and paralogs, is gene conversion^23^. This notion is supported by several other reports suggesting that gene conversion is the most plausible mechanism to explain the evolution for these gene clusters. However, the nucleotide sequences are almost never identical (presence of synonymous mutations) and thus gene conversion alone cannot fully explain the high sequence conservation. To shed light to this issue, we performed pairwise comparisons between paralogous and orthologous sequences using a sliding-window analysis to identify gene regions that are subject to recombination.

**Figure 2:**
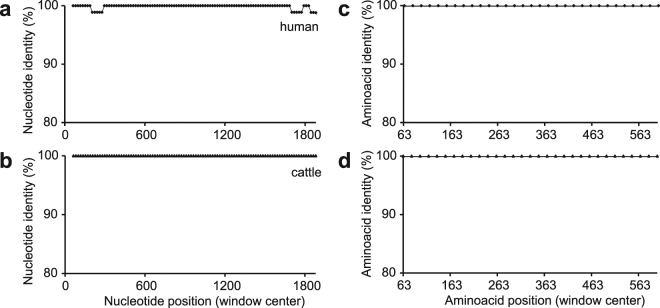
HspA1A and HspA1B genes are highly conserved at both the nucleotide (**a**,**b**) and amino acid (**c**,**d**) levels. Pairwise sequence alignments between human **(a,c)** and cattle **(b,d)** HspA1A and HspA1B sequences over sliding windows were performed using SWAAP (v.1.0.3).

We first calculated the nucleotide and amino acid identities between HspA1A and HspA1B in all species (Fig. 2; Supplementary Fig. S2). These analyses revealed the presence of large genic regions, ranging from 50–100% of the gene sequence, which are identical at both the nucleotide and amino acid levels. Additionally, each species shows a completely different pattern of conservation. These results suggest that in addition to purifying selection, recombination in the form of gene conversion is also playing a role in the evolution of the *HspA1A-1B* gene clusters. Differently, the same analyses comparing HspA1A (or 1B) and HspA1L sequences reveals a pattern consistent with only purifying selection, because in all comparisons the amino acid identity is higher than the nucleotide identity (Supplementary Fig. S3).

**Figure 3:**
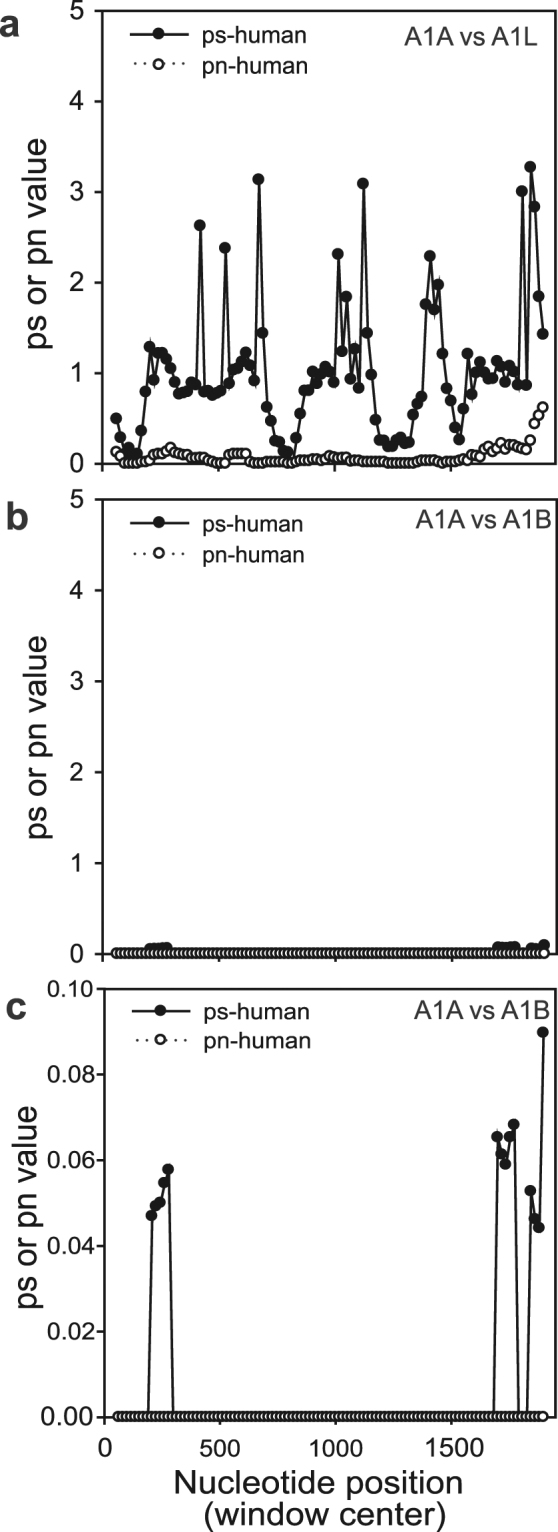
The values of synonymous distances (ps) are 10 to 30 times higher between HspA1A and HspA1L (**a**) than between HspA1A and HspA1AB (**b,c**). Synonymous/non-synonymous substitutions over sliding windows were performed using SWAAP (v.1.0.3) using the modified Nei-Gojobori method. b and c are the same data with different y axes scaling.

The differential action of both purifying selection and gene conversion is also exemplified by analyses of synonymous and non-synonymous substitutions over the length of the *HspA1*-cluster genes (Fig. 3 and Supplementary Fig. S4). First, the number of synonymous substitutions and synonymous distance (ps) values between *HspA1A* and *HspA1L* are 10 to 30 times higher than their equivalent values between *HspA1A* and *HspA1AB* (Fig. 3 and Supplementary Fig. S4). Second, in all comparisons, each species shows a discrete pattern of both synonymous and non-synonymous distance (pn) values (Supplementary Fig. S4). Third, when the *HspA1A* and *HspA1B* sequences are compared, only a few species have any non-synonymous substitutions while most species have multiple synonymous substitutions. In the comparisons between *HspA1A* (or *1B*) and *HspA1L* several non-synonymous substitutions were observed (Supplementary Fig. S4). Some of these substitutions are found at homologous regions, suggesting that the changes occurred early during their diversification and remained conserved (Supplementary Figs S3,S4). Fourth, some species lack substitutions either in their whole or in large parts of their genic region. Collectively, these data strongly support the notion that the *HspA1A*-*1B* gene cluster is evolving under both purifying selection and gene conversion-like mechanisms, and that the strength and frequency of both mechanisms is different between paralogs and orthologs.

### Microevolution of the *HSPA1A* and *HSPA1B* genes in humans

To determine whether the evolutionary patterns observed between species are also detected within a single species and its populations, we analyzed the evolution of the human *HSPA1A-HSPA1B-HSPA1L* genes using SNPs from the 1000-Genomes Database and the Exome Aggregate Consortium (ExAC).

#### Polymorphic sites

In a total genomic region of 20,636 bp (human chromosome 6: 31777396–31798031, *HSPA1A*, *HSPA1B*, and *HSPA1L* genes plus 1Kb upstream of each gene), we found 248 polymorphic sites in the 1000-Genomes dataset. Of these, 45 sites were shared between *HSPA1A* and *HSPA1L* because these genes are located one next to the other in a head-to-head orientation. Their genomic coordinates overlap by 147 bp, which increases to 1,147 bp, when 1Kb is added upstream to either gene. All 248 polymorphic sites within the 1000-Genomes dataset corresponded to bi-allelic SNPs (Table 3). Next, we analyzed approximately the same genomic region (chromosome 6: 31777774–31797705, 19,930 bp) in the ExAC database and found 440 polymorphic sites. This number is approximately 1.8 times larger than the number of sites found in the 1000-Genomes dataset, which is approximately 4% the size of the ExAC dataset. It is important to note here that the difference in SNPs between the two datasets is a conservative estimate for intron-bearing genes, because the ExAC dataset contains mainly exome sequences, thus missing most of the intronic variation. Of the 440 polymorphic sites, 400 were sites with bi-allelic SNPs, and 40 sites with multi-allelic SNPs (seven tri-allelic in *HSPA1L*, 13 tri-allelic in *HSPA1B*, and 17 tri-allelic and three tetra-allelic shared between *HSPA1A* and *HSPA1L*). Additionally, of these 440 polymorphic sites 281 were shared between *HSPA1A* and *HSPA1L* (261 bi-allelic, 20 multi-allelic).

**Table 3:** Polymorphic sites and density for the three *HSPA1* and five neighboring genes reveal that HSPA1A and HSPA1B have low mutational loads. These data are derived from the 1000-Genomes (1000 g) and ExAC datasets. (SNP Density = SNP N/Region Length).

Gene	Region Length (bp)	SNP sites (1000 g)		SNP sites (ExAC)		Density Ratio (ExAC/1000 g)
		N	Density^#^	N	Density	
HSPA1A	2432	39	0.016	46	0.019	1.2
HSPA1B	2519	46	0.018	83	0.033	1.8
HSPA1L	6041	153	0.025	307	0.051	2.0
VARS	18434	410	0.022	961	0.052	2.3
LSM2	9587	228	0.024	114	0.012	0.5
C6orf48	5155	160	0.031	223	0.043	1.4
NEU	5246	85	0.016	285	0.054	3.4
SLC44A4	15853	422	0.027	647	0.041	1.5

Because gene length accounts for ~80% of variation in the number of SNPs across genes, we calculated SNP densities for intergene comparisons. The mean SNP density of both *HSPA1A* and *HSPA1B* was low compared to *HSPA1L* or the five neighboring genes (Table 3), suggesting a lower mutational load for the former genes. However, the mean SNP densities between *HSPA1A* and *HSPA1B* genes (mean SNP density = 0.017) and the six adjacent genes (mean SNP density = 0.024) differ marginally (ANOVA, *p-value* = 0.05). In the ExAC dataset, the *HSPA1A* and *HSPA1B* densities increased by 1.2 and 1.8 times, respectively, while the remaining neighboring genes had fold changes ≤3 (Table 3). The ExAC polymorphic sites number is conservative for the intron-bearing genes (lower limit) because ExAC database is missing most of the intron sequences. To better compare the number of polymorphic sites to the intronless *HSPA1A* and *1B*, we focused on differences in the number of synonymous and non-synonymous (coding) sites. This analysis verified the observed patterns and revealed that the SNP density ratios for *HSPA1A* and *1B* are the lowest in the region (Supplementary Table S3).

#### SNP type distribution

We next sought to investigate the distribution of the different SNP types between the *HSPA1* genes and their neighbors. This analysis revealed that in the intronless *HSPA1A* and *HSPA1B*, the majority of SNPs lie in the 3′ and 5′UTR region, and the most common SNP type is synonymous (Table 4). In the six neighboring genes, which have one or more introns, the majority of the SNPs lie in the intron(s) and 5′ or 3′UTR, and most coding SNPs are nonsynonymous (Table 4). The distribution of SNP types between *HSPA1A* and *HSPA1B* and the neighboring genes in the region is significantly different (Table 4, Pearson p-value < 0.0001). The distribution of SNP types remains significantly different with the inclusion of *HSPA1L* in the *HSPA1* cluster (Supplementary Table S4; Pearson p-value < 0.0001). Similar patterns were observed in the ExAC data, which lack most of the intronic sequences (Supplementary Table S4).

**Table 4:** The distribution of SNP type is significantly different between the *HSPA1* genes and their neighboring genes. This analysis was performed using the 1000-Genomes dataset.

Count Row %	3′URT	5′UTR	CDS_other	intron	missense	synonymous	Total
HSPA1A	7	16	0	0	5	11	39
	17.95	41.03	0.00	0.00	12.82	28.21	
HSPA1B	11	18	0	0	7	10	46
	23.91	39.13	0.00	0.00	15.22	21.74	
HSPA1L	6	26	2	60	37	22	153
	3.92	16.99	1.31	39.22	24.18	14.38	
C6orf48	7	17	2	125	9	1	161
	4.35	10.56	1.24	77.64	5.59	0.62	
LSM2	9	10	1	206	0	2	228
	3.95	4.39	0.44	90.35	0.00	0.88	
NEU	16	7	2	36	12	12	85
	18.82	8.24	2.35	42.35	14.12	14.12	
SLC44A4	11	0	11	339	42	19	422
	2.61	0.00	2.61	80.33	9.95	4.50	
VARS	0	0	10	309	56	35	410
	0.00	0.00	2.44	75.37	13.66	8.54	
Total	67	94	28	1075	168	112	1544
	4.34	6.09	1.81	69.62	10.88	7.25	
			Test	ChiSquare	Prob>ChiSquare		
			Likelihood Ratio	628.751	<0.0001		
			Pearson	627.218	<0.0001		

**Figure 4:**
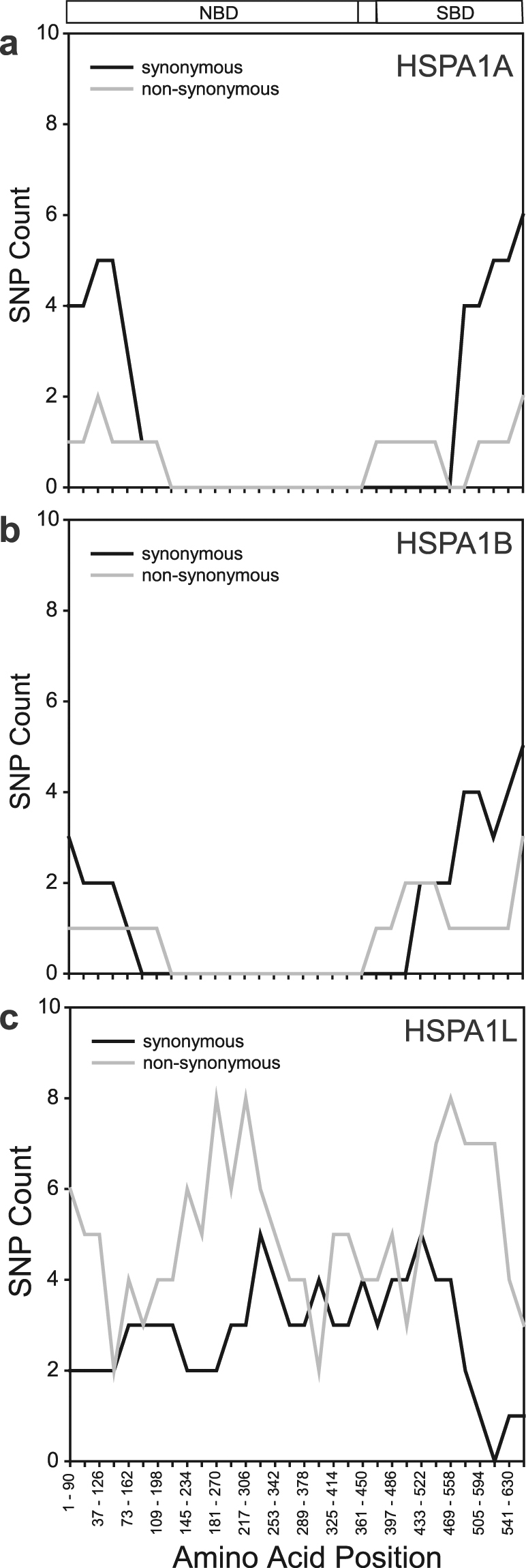
The distribution of SNPs is drastically different between the three HSPA1 genes. Synonymous and non-synonymous SNPs were mapped along the amino acid sequence of (**a**) HSPA1A, (**b**) HSPA1B, and (**c**) HSPA1L using sliding window analysis. The domain organization of Hsp70s (from)^61^ is depicted on the top: NBD = nucleotide binding domain; SBD = substrate binding domain.

We also analyzed the distribution of SNPs in the three *HSPA1* genes using a sliding window to detect the position of these mutations over the length of the sequence (Fig. 4). This analysis revealed that both *HSPA1A* and *HSPA1B* have mutations only at the far N- and C-termini of the encoded protein. This result suggests that more than 80% of positions in both *HSPA1A* and *HSPA1B* have no mutations, which could imply sequence homogenization via gene conversion. Differently *HSPA1L*, which is not predicted to recombine with neither *HSPA1A* nor *HSPA1B*, contains SNPs throughout the length of the gene (Fig. 4). These patterns hold true even when analyzing the ExAC dataset (Supplementary Fig. S5).

#### SNP frequencies

To examine whether the minor allele frequency (MAF) is different between the *HSPA1* cluster and its surrounding genes, we classified SNP frequencies in three categories: (a) rare (MAF <0.005), (b) low frequency (0.005≤ MAF <0.05), and (c) common (MAF ≥0.05). Overall, most (74.35%) of the SNPs in *HSPA1* genes were rare, whereas 19.13% and 6.52% were of low frequency and common, respectively. Similar MAFs were found in the five surrounding genes (Table 5, Pearson *p*-values = 0.1). In the ExAC dataset, SNPs in both *HSPA1* and neighboring genes were mostly rare (95.4% and 97.6%, respectively). The MAF distributions varied significantly between *HSPA1* and neighboring genes, yet the effect sizes of the differences were small (Supplementary Table S5).

**Table 5:** The Minor Allele Frequency (MAF) between the *HSPA1* cluster and its surrounding genes is not significantly different. This analysis was performed using the 1000-Genomes dataset.

Count Col %	HSPA1 genes	Surrounding genes	Total
Common	15	98	113
	6.52	7.66	
Low-freq	44	176	220
	19.13	13.75	
Rare	171	1006	1177
	74.35	78.59	
Total	230	1280	1510
Test	ChiSquare	Prob>ChiSquare	
Likelihood Ratio	4.393	0.11	
Pearson	4.660	0.1	

To examine whether the SNP frequencies differ between the three *HSPA1* genes we compared their MAF categories in both the 1000-Genomes and ExAC datasets. Both datasets showed that in all three genes most of the SNPs are rare, with *HSPA1B* containing the highest number of low-frequency SNPs, and *HSPA1A* and *HSPA1B* containing more common SNPs than *HSPA1L* (Row % in Supplementary Table S6).

We also determined whether the *HSPA1* genes contain any population specific SNPs. This analysis revealed that after excluding rare SNPs, only four of them were population specific: rs2607018, rs114406544, rs34791928, and rs34372373, all being transitions and found at the 5′UTR of *HSPA1B* (AFR population), and 3′UTR (AFR population), intron (EAS population), and exon (synonymous) (AFR population) of *HSPA1L*, respectively.

We next determined the relationship between MAF and the different human populations. The mean MAF (UTRs and CDS) in all populations was 0.038 (CI = 0.03–0.05) in the *HSPA1* cluster. Within each gene, the mean gene MAFs ranged from 0.02–0.09 (Table 6). In all populations, *HSPA1A* had the highest MAF values, followed by *HSPA1B*, and *HSPA1L* (Table 6). Furthermore, the distribution of MAFs within each population was highly (positively) skewed, and the MAFs were not normally distributed (goodness-of-fit test *p-value* < 0.001). The mean MAFs were significantly different among the AFR and EUR and AFR and EAS populations (Kruskal-Wallis test, χ^2^ *p-value* = 0.012 and 0.007, respectively) (data not shown).

**Table 6:** The relationship between MAF and the different human populations was analyzed for each *HSPA1* gene using the 1000-Genomes dataset.

Population	Level	SNP count	Mean MAF	Std Error	Lower 95%	Upper 95%
AFR	HSPA1A	37	0.09	0.03	0.04	0.14
	HSPA1B	43	0.06	0.03	0.01	0.10
	HSPA1L	94	0.03	0.02	−0.01	0.07
SAS	HSPA1A	37	0.06	0.02	0.02	0.11
	HSPA1B	43	0.04	0.02	0.00	0.08
	HSPA1L	94	0.02	0.02	−0.01	0.05
EAS	HSPA1A	37	0.06	0.02	0.02	0.10
	HSPA1B	43	0.04	0.02	0.00	0.07
	HSPA1L	94	0.02	0.01	−0.01	0.05
EUR	HSPA1A	37	0.06	0.02	0.02	0.10
	HSPA1B	43	0.04	0.02	0.00	0.08
	HSPA1L	94	0.02	0.01	−0.01	0.05
AME	HSPA1A	37	0.07	0.02	0.02	0.11
	HSPA1B	43	0.04	0.02	0.00	0.08
	HSPA1L	94	0.02	0.02	−0.01	0.06
ALL	HSPA1A	37	0.07	0.02	0.10	0.04
	HSPA1B	43	0.04	0.01	0.06	0.02
	HSPA1L	94	0.02	0.01	0.03	0.01

### Functional consequences of SNPs

We selected six SNPs (Supplementary Table S7) that satisfied most of our criteria (see Materials and Methods section) and performed three types of assays that assessed the impact of these mutations on protein structure and function. In addition to the WT protein, which served as a positive control, a mutation known to minimize ATP hydrolysis (K71A) was also used as a negative control.

#### Protein stability

We first determined whether a particular mutation affects protein stability by measuring whether it alters the melting temperature (Tm) of HSPA1A. The results of this assay showed that all mutations tested alter the Tm of HSPA1A, and thus are predicted to alter its stability (Table 7 and Supplementary Fig. S6). In particular, the mutations S16P, S16Y, I74T, and F592S decreased HSPA1A’s Tm, while the R36C and I480N increased it. Although the effect of these changes in protein Tm is unknown in a physiological environment, we speculate that these relatively small changes (between 0.5 and 3 °C) have subtle consequences.

**Table 7:** Melting temperature (Tm) of the wild type and mutated HSPA1A variants calculated by fitting the Thermal Shift Assay data using the Boltzmann equation. The values are presented as averages of three independent experiments. Statistical significance was determined by Student’s *t* test. A *p*-value < 0.05 was considered statistically significant. SD: standard deviation.

Protein	Tm (°C)	SD	*t* test (*p*-value vs WT)
WT	43.49	0.1485	na
K71A	46.79	0.1464	<0.0001
S16P	43.08	0.1092	0.0188
S16Y	41.32	0.2594	0.0002
R36C	44.24	0.2604	0.0125
I74T	40.65	0.1025	<0.0001
I480N	43.59	0.2940	0.6276
F592S	40.37	0.3196	0.0001

#### Binding to nucleotides

**Figure 5:**
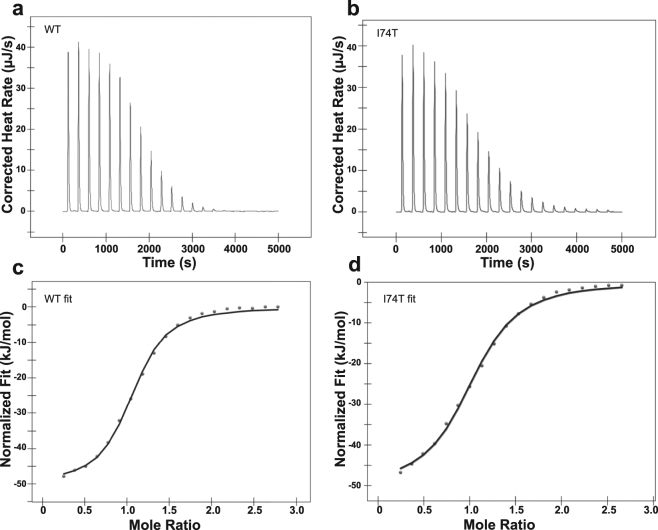
The wild type (WT) and mutated (I74T) HspA1A variant bind similarly to ATP. Representative Isothermal Titration Calorimetry (ITC) assays using purified recombinant HspA1A proteins and ATP. (**a,b**) Represent the ITC raw data for 20 automatic injections of ATP into the sample cell containing either WT or mutated HspA1A. (**c,d**) Represent ITC binding curves obtained for the interaction between ATP and HspA1A. The data shown are representative of three independent experiments.

We next tested whether these mutations alter the binding of HSPA1A to ATP and ADP using isothermal titration calorimetry (ITC). Our results suggest that the effects of these mutations are small (Fig. 5), but reveal some interesting findings about particular mutations, which can be summarized as follows (Supplementary Fig. S7 and Supplementary Table S8). First, most variants bind to ATP with similar affinity to the WT with the exception of the S16P mutant. Furthermore, analyses of the enthalpy (delta H) and the entropy (delta S) of the interactions suggest that the different types of molecular forces, e.g., electrostatic and hydrophobic, that govern HSPA1A’s binding to ATP remain relatively unchanged. Second, the affinity for ADP is only altered in the case of the S16P and R36C mutations (Supplementary Table S8). The enthalpy and entropy of the interactions also remain relatively unchanged. These small changes suggest that the nucleotide binding function of HSPA1A remains conserved.

#### Protein localization

**Figure 6:**
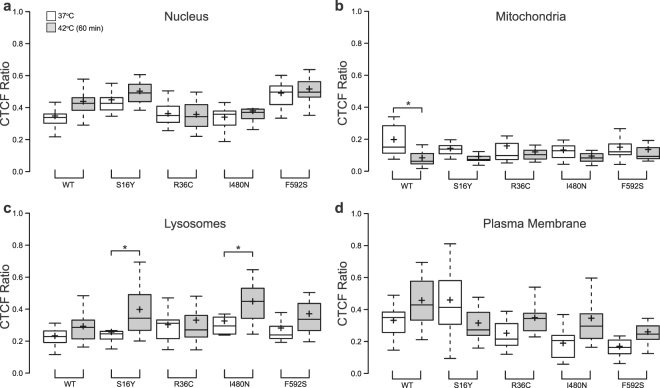
The mutated HspA1A variants have similar localization as compared to the WT protein. Quantification of the corrected total cell fluorescence (CTCF) as a ratio between the total GFP-HspA1A fluorescence of particular intracellular compartment [(**a**) nucleus, (**b**) mitochondria, (**c**) lysosome, and (**d**) plasma membrane] and the rest of the cell. White bars indicate cells that maintained at 37 °C and grey bars indicate cells that were stressed at 42 °C for 60 min. Center lines show the medians; box limits indicate the 25th and 75th percentiles as determined by R software (http://shiny.chemgrid.org/boxplotr/); whiskers extend 1.5 times the interquartile range from the 25th and 75th percentiles; crosses represent sample means. The experiment was repeated three times and in total 15 cells were used to generate each boxplot. Star (*) denotes statistical significance. The *p*-values of the student Holm test were: S16Y-37 vs 42 = 0.04; F592S-37 vs 42 = 0.01 and WT-37 vs 42 = 0.01.

We next tested the effect of these mutations on HspA1A misfolding and localization in human cells before and after a mild heat shock (42 °C). Our results reveal that none of these mutations induce protein misfolding and aggregation (Fig. 6 and Supplementary Fig. S8). Furthermore, the mutated HSPA1A variants have very similar localization with the WT protein at 37 °C (Fig. 6; white boxplots), with a few exceptions. Specifically, in the lysosomes the CTCF ratio of I480N and F592S was 40% (*p-value* = 0.05) and 34% (*p-value* = 0.01) lower than the WT protein, and in the nucleus the CTCF ratio of S16Y and F592S was approximately 29% (*p-value* = 0.02) and 40% (*p-value* < 0.001) higher than the WT. Additionally, the pattern of heat shock-induced translocation for HSPA1A seemed to be retained. For example, in all cases, heat-shock caused HSPA1A to translocate to the nucleus or the plasma membrane and move away from the mitochondria. However, some mutations resulted in a few small differences in HSPA1A’s intracellular localization (Fig. 6 and Supplementary Fig. S8). For example, the R36C and I480N showed less translocation in the nucleus, the S16Y and I480N showed an increase in the lysosomes, and the S16Y did not show movement towards the plasma membrane.

## Discussion

The concomitant action of multiple mechanisms resulted in different modes of evolution in the Hsp70 protein family^7,8^. Contrasting evolutionary mechanisms including concerted, divergent, and convergent, resulted in sub- and neo-functionalization and elicited both functional promiscuity and specificity^5,7–9,13,14,18^.

Our results on the evolution of the heat-inducible *hsp70* genes in mammals suggest an additional evolutionary phenomenon, in which gene diversification and sequence conservation have been shaped by the concurrent action of purifying selection and gene-conversion. Furthermore, our results reveal that the modes of evolution observed between species are also occurring within humans despite the very short time of human evolution. Further support for the action of purifying selection in such a short period of time derives from the functional characterization of specific mutations, which revealed subtle differences in protein stability and intracellular localization. Our findings support a scenario that explains the evolution of the *HspA1* cluster in mammals. According to this scenario, the very first mammals had both *HspA1L* and the ancestral *HspA1A/B* gene within their MHC-III region. Later, in the dawn of placental mammals a single gene duplication event gave rise to the *HspA1A-1B* gene cluster and all three genes remained within the MHC-III region during the mammalian radiation. The intraspecies clustering of all *HspA1A* and *HspA1B* genes studied suggests the action of gene conversion, and the presence of synonymous mutations the action of purifying selection^9,15^. Our scenario also suggests that *HspA1L* genes evolved under a divergent model of evolution under purifying selection. The effect of selection appears to be very strong because the substitutions that occurred early during paralog diversification have been conserved after multiple speciation events. Furthermore, our scenario suggests that in all species the *HspA1L* and *HspA1A-1B* genes evolved independently accumulating discreet patterns of both synonymous and non-synonymous mutations.

According to our findings a single evolutionary mechanism, namely purifying selection or gene conversion, fails to fully explain the observed patterns of conservation between *HspA1A* and *HspA1B* genes^9,15,24^. Furthermore, we speculate that the gene-conversion events occurred very early after the gene duplication resulted in the *HspA1A-HspA1B* cluster. Additionally, gene conversion continued to frequently homogenize the sequences, because there are many regions of the genes that are identical even at the nucleotide level in specific groups of species. The presence of frequent meiotic recombination events is also supported by the fact that the length of the recombined sequence is not identical between different species, but rather it varies in length as expected by its molecular mechanism in yeast and mammalian cells^25–27^. The frequent gene-conversion events are also supported by the finding that both the number of substitutions and the values of synonymous and non-synonymous distances between *HspA1A* and *HspA1L* are considerably higher than their equivalent values between *HspA1A* and *HspA1B*. This notion is supported by investigations in flies in which several frequent events of recombination were proposed^15^. However, differently from previous studies^28–33^, our findings suggest that these recombination events were complemented by the action of purifying selection. Furthermore, even in cases in which gene conversion and purifying selection have been used to explain the evolution of a particular gene, e.g., opsin or trypsin genes in primates^32,33^, the two mechanisms were found to compete than to complement.

In addition to the interspecies evolutionary analyses, our analyses of the microevolutionary mechanisms within humans further support the presence of both purifying selection and gene conversion acting on the *HSPA1A-B* gene cluster. Both the total counts as well as the polymorphism densities show that human *HSPA1A* and *HSPA1B* have a very small number of mutations especially in their coding sequence. This is particularly true when these values are compared to *HSPA1L* and other neighboring genes (Table 4 and Supplementary Table S4). The uniqueness of *HSPA1A*-*1B* in which gene conversion and purifying selection act together stems also from the finding that the vast majority of polymorphisms lie in the UTR regions, and their most common SNP type in the coding region is synonymous. Both of these findings are different when compared to other genes and findings from the whole genome^34^. Furthermore, polymorphism analysis in all three *HSPA1* genes revealed that the majority of the *HSPA1A-1B* gene length contains no mutations, while the *HSPA1L* gene sequence has several mutations throughout its gene length. Similar patterns of genetic variation were also observed in the ExAC dataset^35^, which is 24 times larger than the 1000-Genomes dataset (Fig. 4 and Supplementary Fig. S5). Although we did not find shared polymorphisms (a strong indicator of gene conversion^23^, we speculate that this observation may be related to the fact that *HSPA1A* and *HSPA1B* are able to recombine with each other resulting in homogenized sequences, while *HSPA1L* does not. If this supposition is correct, then it further supports our suggestion of frequent gene conversion.

Similarly to the rest of the genome^34^ and their neighboring genes, *HSPA1A* and *1B* contain mainly rare mutations, although both have more common mutations than *HSPA1L* (Table 5 and Supplementary Tables S5 and S6). Moreover, only *HSPA1L* contains a few population specific SNPs and in all populations *HSPA1A* has the highest MAF values (Table 6). Together these findings exemplify the function of purifying selection, but also reveal that the mutations accumulated early in the evolution of *HSPA1A* and *1B* in humans remained during human radiations. Further support for the action of purifying selection stems from the distribution of SNP frequencies in different human populations, which deviates from normality and is positively skewed towards rare UTR and synonymous SNPs.

All the above observations point to strong negative selection acting on both *HSPA1A* and *1B* genes. This notion is supported by analysis that failed to identify any polymorphic site under positive or balancing selection (Supplementary Tables S9 and S10). The latter observation may be suggestive of neutral evolution driven by drift^36^. However, drift cannot explain the specific localization of polymorphisms mainly at the UTRs of *HSPA1A-1B*. Additionally, the finding that all the known functional amino acids remain conserved both within human populations and between species is highly suggestive of purifying selection. We wondered whether background selection^37^ could explain the observed patterns of conservation throughout the *HSPA1A-1B* genes. Based on this notion, neutral mutations (mutations on sites of unknown function) will be lost as a result of purifying selection against linked deleterious mutations on sites that are functionally important^37–41^. However, background selection alone fails to explain the accumulation of mutations at the UTRs of *HSPA1A-1B* and the lack of mutations in the central part of the genes. Furthermore, background selection assumes that low levels of recombination will result in low levels of genetic variation. However, based on our results *HSPA1A* and *HSPA1B* are frequently recombined via gene conversion, thus, background selection may not be the most plausible explanation of the observed conservation and instead suggest the simultaneous action of both purifying selection and gene-conversion for the evolution of *HSPA1A* and *HSPA1B*.

The functional assays also strongly support the notion that purifying selection is acting on *HSPA1A* and *1B* genes, because even the most chemically radical mutations observed did not result in drastic changes in protein stability or localization, or in loss-of-function. Therefore, we believe that the observed functional changes are the results of negative selection that manifests its effects even at the very short time of human evolution. Several reports revealed the direct relationship between inducible Hsp70s and thermotolerance, survival after stress, and fitness^17,42–46^ and our findings in mammals are in accordance with the predictions of these studies. Our results also support the concurrent action of both purifying selection and gene conversion in mammalian Hsp70s, mechanisms that have been also shown to direct the evolution of their homologs in flies and nematodes^9,15^. Although HspA1A and HspA1B knockout mice are viable, they are heat-sensitive^4,44,45^. Similarly, knockout flies^47^ are viable and fertile, but have issues recovering from heat shock, and show developmental delays. These findings suggest that inducible Hsp70s have distinct biological functions as compared to their constitutively expressed homologs. This notion is further supported by the fact that individual Hsp70 proteins have additional chaperone-independent and context-specific functions^4,5,48^. Therefore, selection may be acting to preserve not only the primary function but also other less well-studied secondary functions of the molecules, like lipid binding and cell signaling.

There are a lot of unanswered questions on the evolution and natural variation patterns of Hsp70s and other molecules functioning in the CSR. Nevertheless, our results from inter- and intraspecies phylogenetic and genomic comparisons coupled with functional studies demonstrate how selection and gene conversion combine across evolutionary time to reduce the impact of mutations on function. These findings provide insights to a paradigm of strong and continuing genetic mechanisms and evolutionary forces that conserve one of the most critical cell components driving the ability of all organisms to survive stress and thrive.

## Materials and Methods

### Sequence collection and interspecies analyses

To determine the origin and evolution of *HSPA1A* and *HSPA1B*, protein sequences were collected from the National Center for Biotechnology Information (NCBI) mammalian reference sequence protein database using protein-BLAST^49^ searches with the human HSPA1A, HSPA1B, and HSPA1L as queries and default parameters. The collected proteins from this initial search were mapped back to the corresponding mammalian genome sequence to identify the genomic organization of the genes and ensure collection of all three Hsp70 genes. Based on this search, nucleotide sequences (genomic and coding) were also collected for the corresponding genes identified above. Furthermore, at least two neighbor genes from the 5′ and 3′ of the Hsp70 cluster were also collected and were used to determine conservation of synteny. Although there are more than 100 mammalian genomic sequences at different levels of completion available at the NCBI website, we identified 30 species in which the region and all three Hsp70 genes were complete (Supplementary Fig. 1). Out of these we selected nine representative species that were further analyzed (Fig. 1).

The collected sequences were aligned with CLUSTALW in MEGA 6.0^50^ using default parameters, and manually corrected in BioEdit. Pairwise sequence alignments and synonymous/non-synonymous substitutions over sliding windows were performed using SWAAP (v.1.0.3). Nucleotide and amino acid pairwise identities, as well as synonymous and non-synonymous distances (modified Nei-Gojobori method) were performed with MEGA 6.0.

Maximum-likelihood was used to find the best model of evolution (MEGA 6.0)^50^ for the protein sequences. Based on the Bayesian Information Criterion (BIC) the substitution pattern was best described by the JTT model with corrections for non-uniform evolutionary rates among sites (+G; gamma value = 0.22). Phylogenetic trees were generated using the neighbor-joining (NJ), Maximum-likelihood (ML), and maximum parsimony algorithms as implemented in MEGA 6.0. All three methods provided similar topologies for the major branches (contain three numbers). One thousand bootstrap pseudo-replicates were used to test the reliability of the inferred trees. All positions containing gaps were eliminated and there were a total of 637 positions in the final dataset.

### Collection and analysis of SNPs

We next sought to test whether the patterns of nucleotide and amino acid conservation and diversification observed between different mammals were also observed within a single species. In this case, we capitalized on the presence of several thousand genomes or exomes from humans and collected single nucleotide polymorphisms (SNPs) from Ensembl’s 1000-Genomes browser (http://grch37.ensembl.org/Homo_sapiens/Info/Index) using all available data present within the 1000-Genomes database (phase 3)^34^. Similarly, we collected data for *HSPA1L* and five surrounding genes (*C6orf48, LSM2, NEU, SLC44A4*, and *VARS*) for comparative purposes. The data were filtered to include SNPs found only in the gene region and in the 1000-Genomes project. For each *HSPA1* and five neighboring genes, SNP data were also collected from the ExAC database (August 2016)^35^. This dataset contains variants found in the exomes of 60,706 unrelated individuals from disease-specific and population genetic studies and is 24.24 times larger than the 1000-Genomes dataset. The patterns of SNP density within the *HSPA1* genes and surrounding genes were also calculated by dividing the total number of SNPs within a given region by that region’s sequence length in bp.

To collect each *HSPA1* gene’s available allele frequencies of natural extant variation we used the 1000-Genomes database (http://www.1000genomes.org/home, Phase 3). Phase 3 contains >84 × 10^6^ variants from 2,504 individuals sampled from 26 populations^34^. We collected the allele frequency of each chromosomal region using the ‘allele frequency calculator’ tool (http://grch37.ensembl.org/Homo_sapiens/Tools/AlleleFrequency). A detailed documentation of the tool can be found at http://www.1000genomes.org/allele-frequency-calculator-documentation. After collection of data for all genes, two positions with >1 alleles listed/entry (one in *HSPA1L* and one in *HSPA1B*) were discarded.

### Tests for selection

To understand the contribution of selection in shaping population variation, we examined whether *HSPA1A, HSPA1B*, and *HSPA1L* human polymorphisms deviate from neutrality. For this we used two F_ST_ outlier tests: FDIST^51^ and BayeScan^52^. Both methods make assumptions about the demographic history of the samples. FDIST simulates the island model as a null distribution to test for significance, whereas the Bayesian method assumes that the samples have diverged independently from a common ancestor, and utilizes the multinomial Dirichlet distribution to describe the gene frequencies under a wide range of demographic models.

To perform the FDIST calculations we used the LOSITAN workbench^53^. Gene vcf files were retrieved from the 1000-Genomes ftp site (ftp://ftp.1000genomes.ebi.ac.uk//vol1/ftp/technical/reference/phase2_reference_assembly_sequence/hs37d5.fa.gz) using tabix tool^54^. Gene vcf files were subsequently transformed to genepop files by using the PGPSpider software^55^. Vcf files were first converted to PGD files and PGD files were then converted to genepop ones. Genepop files were used as input files in LOSITAN. We also used a population file that included the five major 1000-Genomes populations: African, American, European, East Asian, and South Asian. Within LOSITAN, rare polymorphisms (global MAF <0.005) were excluded. We simulated the neutral distribution of F_ST_ with 10^5^ iterations at a significance level P < 0.005. The “neutral” mean F_ST_ option was chosen, confidence intervals were set at 0.99, false discovery rate at 0.01, subsample size at 50, and the mutational model was set at infinite alleles.

To run the Bayesian method, we used BayeScan. Genepop files were converted to BayeScan input files using PGPSpider. The default Markov chain and model parameters were used, except for thinning and pilot runs, which were set at 100 and 200 respectively. Within BayeScan, rare SNPs (global MAF <0.005) were discarded.

### Determination of amino acids of known function

To identify amino acid positions with verified functional output, studies reporting mutagenesis experiments on HSP70 homologs were collected. The data were mined per amino acid position and filtered to include only experiments that determined a functional output e.g., loss-of-function, decrease in binding affinity. Homologs were aligned with the human HSPA1A protein sequence using the Needle pairwise sequence alignment program, which produces a global alignment (EMBOSS; https://www.ebi.ac.uk/Tools/psa/emboss_needle/). The Hsp70 amino acid positions determined to have a known function were cross-referenced with silent and non-synonymous single-nucleotide polymorphisms (SNPs) reported in NCBI’s dbSNP and the 1000-Genomes browser.

### Computational predictions of non-synonymous SNPs

To predict the functional effect of non-synonymous SNPs found within *HSPA1A* and *HSPA1B* genes we used five major criteria. First, the SNPs were categorized based on whether they change an amino acid of known function, because a mutation on a site of established function would most probably have a functional impact. Second, the SNPs were categorized based on whether they occur on a highly conserved amino acid position by determining the amino acid conservation level of each position, because we assumed that highly conserved amino acids would have a higher probability of causing a functional change. Third, the SNPs were classified based on whether the amino acid change was predicted to be radical (different amino acid class and negative or zero scores in both BLOSUM 65 and 80). The rationale of the latter criterion relies on the fact that radical changes may alter the function with a higher probability than non-radical amino acid changes. Fourth, the SNPs were categorized based on whether a particular mutation is predicted to alter the local conformation or the molecule surface by generating three-dimensional models of the wild-type (WT) and mutated proteins. And fifth, the SNPs were categorized based on the outputs of polyphen, shift, and SNAP^2,56,57^.

### Generation of mutated recombinant clones, proteins, protein purification

The cDNA clone containing the *hspA1A* gene sequence, accession number BC054782 was used to generate the recombinant clones used in this study. The coding sequence was subcloned into the pet-22b vector for protein production in bacteria as described in McCallister *et al*.^5^. For expression in mammalian cells, the gene was subcloned into the pEGFP-C2 vector using directional cloning after PCR amplification and restriction digest (see Supplementary Table S7 for primer sequences and^5^ for complete protocol). Site directed mutagenesis using long-PCR amplification followed by *Dpn*I digestion was used to generate the mutated Hsp70 variants. Specifically, 10 ng of plasmid DNA were mixed with 125 ng each primer (Supplementary Table S7) in a 50 µl reaction containing 1 µl DMSO, 5 µl Buffer, 1 µl of 10 mM dNTPs, and 1 µl (2.5 units) of native PFU polymerase (Agilent). The whole plasmid was then amplified using the following conditions: 5 min at 95 °C; (30 sec at 95 °C; 1 min at 60 °C; 15 min at 72 °C) cycle repeated 16 times; 15 min at 72 °C. After sequence verification, the mutated and wild type (WT) constructs were used to generate and purify recombinant proteins exactly as described in^5,58^.

### Protein activity tests

To test the effects of the mutations on the structure and function of HSPA1A, we determined whether they affect protein stability and binding to nucleotides (ATP, ADP). All experiments were repeated at least three times using different batches of recombinant proteins. Statistical significance was determined by an unpaired t-test. A *p*-value < 0.05 was considered statistically significant.

To determine the effect of naturally occurring mutations on protein stability the Thermal Shift Assay (TSA; Thermo Scientific) was employed and protein stability was determined as a function of the protein melting temperature (Tm). In this assay, 5 µM of each protein was mixed with 5 μl of Protein Thermal Shift™ Buffer (Thermo Scientific) and 2.5 µl diluted Protein Thermal Shift™ Day (8×) in 20 µl total volume. The mixture was incubated in CFX96 Real-Time PCR Detection System (BioRad) under continuous ramp mode, from 16–95 °C, using a 0.05 °C/s ramp rate. To calculate the Tm, the data were plotted and fitted to the Boltzmann equation using SigmaPlot v10. The Tm values obtained were averaged per protein and the standard deviation was calculated.

We next employed Isothermal Titration Calorimetry (ITC) to determine whether mutated variants of HSPA1A bind to ATP and ADP similarly to the WT protein. ITC measurements were performed at 25 °C with a NanoITC calorimeter (TA Instruments, New Castle, DE). For these experiments, 4 µM of each protein were diluted in 250 μl (final volume) of a buffer containing 20 mM Tris (pH 7.5), 40 mM NaCl, 4 mM MgAc_2_, and 0.5 mM EDTA. The ligands, 8 mM ATP or ADP were diluted in a 60 μl of the same buffer. After sample degassing, the protein was loaded into the cell and the ligands in the titration syringe. For each injection, a 2.5 µl portion of each ligand was injected into the cell, at 240 second intervals. The data were processed with the NanoITC software (NanoAnalyze Software v3.7.0).

### Cell culture, transfection, confocal microscopy, and image analysis

To determine whether the mutations result in HSPA1A misfolding or alter its intracellular localization we transfected mutated and WT GFP-tagged HSPA1A into human cells and visualized the protein using confocal microscopy. The rational of these experiments relies on the notion that if a mutation drastically alters protein structure, this may result in misfolding and improper intracellular localization.

Our focus was to determine the effect of the mutation on the protein and not to study the behavior of cells carrying these mutated HspA1A variants under stress. For this purpose, we used HEK293 cells (which were purchased from ATCC; ATCC^®^ CRL-1573^™^). The cells were maintained in a humidified 5% CO_2_ atmosphere at 37 °C in complete medium consisting of DMEM supplemented with 10% fetal bovine serum, 2 mM L-glutamine, and penicillin-streptomycin. A day before transfection cells were split into 24-well plates at 2.0 × 10^3^ cells/well containing poly-D-lysine treated coverslips. After 18 hours cells were transiently transfected with the WT or mutant HSPA1A construct using the PolyJet *In Vitro* DNA Transfection Reagent (SignaGen) as per the manufacturer’s instructions. Transfection was allowed to continue for 18 hours, and then transfection media was removed and replaced with fresh complete media. At that time cells were either maintained at 37 °C or were placed in a water-bath at 42 °C for 60 minutes. After stress, the nucleus was stained using DAPI (present in the Fluoromount-G mounting media; Southern Biotech), the mitochondria using MitoTracker Red (100 nM; Life Sciences), and the plasma membrane using wheat germ agglutinin-AF555 (500 ng/mL; Life Sciences). The lysosomes were visualized by co-transfecting the HSPA1A variant with the lysosome-associated membrane protein 1 (LAMP1)-mRFPC1 construct [a gift from Walther Mothes (Addgene plasmid # 1817)]. After staining, cells were fixed using freshly prepared solution of 4% paraformaldehyde in complete growth medium. Cells were visualized using a Leica DM IRE2 confocal microscope equipped with a 63 × 1.4 oil objective. Image and co-localization analyses were performed for 15 cells from three independent experiments. Images were analyzed in ImageJ^59^ using the corrected total cell fluorescence (CTCF) method^60^ as a ratio between the total GFP-HspA1A fluorescence of a particular compartment and the rest of the cell. Statistical significance was determined by one way Anova with post-hoc Tukey HSD (honest significant difference) and Holm multiple comparison tests. A *p*-value < 0.05 from both post-hoc tests was considered statistically significant.

### Data and Materials Availability

The datasets and materials generated during the current study are available from the corresponding author on reasonable request.

## Electronic supplementary material

Supplementary Material

## References

[1] Kultz D (2003). Evolution of the cellular stress proteome: from monophyletic origin to ubiquitous function. The Journal of experimental biology.

[2] Kultz D (2005). Molecular and evolutionary basis of the cellular stress response. Annu Rev Physiol.

[3] Lindquist S, Craig EA (1988). The heat-shock proteins. Annu Rev Genet.

[4] Daugaard M, Rohde M, Jaattela M (2007). The heat shock protein 70 family: Highly homologous proteins with overlapping and distinct functions. FEBS Lett.

[5] McCallister C, Siracusa MC, Shirazi F, Chalkia D, Nikolaidis N (2015). Functional diversification and specialization of cytosolic 70-kDa heat shock proteins. Sci Rep.

[6] Richter K, Haslbeck M, Buchner J (2010). The heat shock response: life on the verge of death. Mol Cell.

[7] Boorstein WR, Ziegelhoffer T, Craig EA (1994). Molecular evolution of the HSP70 multigene family. Journal of molecular evolution.

[8] Brocchieri L, Conway de Macario E, Macario A (2008). hsp70 genes in the human genome: Conservation and differentiation patterns predict a wide array of overlapping and specialized functions. BMC Evolutionary Biology.

[9] Nikolaidis N, Nei M (2004). Concerted and nonconcerted evolution of the Hsp70 gene superfamily in two sibling species of nematodes. Mol Biol Evol.

[10] Gupta RS, Singh B (1994). Phylogenetic analysis of 70 kD heat shock protein sequences suggests a chimeric origin for the eukaryotic cell nucleus. Curr Biol.

[11] Kabani M, Martineau CN (2008). Multiple hsp70 isoforms in the eukaryotic cytosol: mere redundancy or functional specificity?. Curr Genomics.

[12] Arispe N, Doh M, Simakova O, Kurganov B, De Maio A (2004). Hsc70 and Hsp70 interact with phosphatidylserine on the surface of PC12 cells resulting in a decrease of viability. FASEB J.

[13] Kourtidis A (2006). Identification of several cytoplasmic HSP70 genes from the Mediterranean mussel (Mytilus galloprovincialis) and their long-term evolution in Mollusca and Metazoa. Journal of molecular evolution.

[14] Krenek S, Schlegel M, Berendonk TU (2013). Convergent evolution of heat-inducibility during subfunctionalization of the Hsp70 gene family. BMC Evol Biol.

[15] Bettencourt BR, Feder ME (2002). Rapid concerted evolution via gene conversion at the Drosophila hsp70 genes. Journal of molecular evolution.

[16] Kudla G, Helwak A, Lipinski L (2004). Gene conversion and GC-content evolution in mammalian Hsp70. Mol Biol Evol.

[17] Bettencourt BR, Feder ME (2001). Hsp70 duplication in the Drosophila melanogaster species group: how and when did two become five?. Mol Biol Evol.

[18] Macario AJ, Brocchieri L, Shenoy AR, Conway de Macario E (2006). Evolution of a protein-folding machine: genomic and evolutionary analyses reveal three lineages of the archaealhsp70(dnaK) gene. Journal of molecular evolution.

[19] Singh R (2006). Reduced heat shock response in human mononuclear cells during aging and its association with polymorphisms in HSP70 genes. Cell Stress Chaperones.

[20] He M (2009). Functional SNPs in HSPA1A gene predict risk of coronary heart disease. PLoS One.

[21] Maugeri N, Radhakrishnan J, Knight JC (2010). Genetic determinants of HSP70 gene expression following heat shock. Hum Mol Genet.

[22] Kumar S, Stecher G, Suleski M, Hedges SB (2017). TimeTree: A Resource for Timelines, Timetrees, and Divergence Times. Mol Biol Evol.

[23] Mansai SP, Innan H (2010). The power of the methods for detecting interlocus gene conversion. Genetics.

[24] Nei M, Rooney AP (2005). Concerted and birth-and-death evolution of multigene families. Annu Rev Genet.

[25] Chen JM, Cooper DN, Chuzhanova N, Ferec C, Patrinos GP (2007). Gene conversion: mechanisms, evolution and human disease. Nat Rev Genet.

[26] Haber JE (1999). DNA recombination: the replication connection. Trends in biochemical sciences.

[27] Gonzalez-Barrera S, Garcia-Rubio M, Aguilera A (2002). Transcription and double-strand breaks induce similar mitotic recombination events in Saccharomyces cerevisiae. Genetics.

[28] Lartillot N (2013). Interaction between Selection and Biased Gene Conversion in Mammalian Protein-Coding Sequence Evolution Revealed by a Phylogenetic Covariance Analysis. Molecular Biology and Evolution.

[29] Verrelli BC, Tishkoff SA (2004). Signatures of selection and gene conversion associated with human color vision variation. Am J Hum Genet.

[30] Ohta T (2010). Gene Conversion and Evolution of Gene Families: An Overview. Genes-Basel.

[31] Yasukochi Y, Satta Y (2015). Molecular Evolution of the CYP2D Subfamily in Primates: Purifying Selection on Substrate Recognition Sites without the Frequent or Long-Tract Gene Conversion. Genome Biology and Evolution.

[32] Hiwatashi T (2011). Gene conversion and purifying selection shape nucleotide variation in gibbon L/M opsin genes. BMC Evol Biol.

[33] Petronella N, Drouin G (2012). Strong purifying selection against gene conversions in the trypsin genes of primates. Hum Genet.

[34] Sudmant PH (2015). An integrated map of structural variation in 2,504 human genomes. Nature.

[35] Lek M (2016). Analysis of protein-coding genetic variation in 60,706 humans. Nature.

[36] Kimura M (1970). The length of time required for a selectively neutral mutant to reach fixation through random frequency drift in a finite population. Genet Res.

[37] Charlesworth B, Morgan MT, Charlesworth D (1993). The effect of deleterious mutations on neutral molecular variation. Genetics.

[38] Stephan W (2010). Genetic hitchhiking versus background selection: the controversy and its implications. Philos Trans R Soc Lond B Biol Sci.

[39] Nachman MW (2001). Single nucleotide polymorphisms and recombination rate in humans. Trends Genet.

[40] Nielsen R (2005). Molecular signatures of natural selection. Annu Rev Genet.

[41] Pool JE, Hellmann I, Jensen JD, Nielsen R (2010). Population genetic inference from genomic sequence variation. Genome Res.

[42] Loeschcke V, Krebs RA, Dahlgaard J, Michalak P (1997). High-temperature stress and the evolution of thermal resistance in Drosophila. EXS.

[43] Krebs RA (1999). A comparison of Hsp70 expression and thermotolerance in adults and larvae of three Drosophila species. Cell Stress Chaperones.

[44] Hunt CR (2004). Genomic instability and enhanced radiosensitivity in Hsp70.1- and Hsp70.3-deficient mice. Mol Cell Biol.

[45] Kim YK (2006). Deletion of the inducible 70-kDa heat shock protein genes in mice impairs cardiac contractile function and calcium handling associated with hypertrophy. Circulation.

[46] Feder ME, Krebs RA (1997). Ecological and evolutionary physiology of heat shock proteins and the stress response in Drosophila: complementary insights from genetic engineering and natural variation. EXS.

[47] Gong WJ, Golic KG (2006). Loss of Hsp70 in Drosophila is pleiotropic, with effects on thermotolerance, recovery from heat shock and neurodegeneration. Genetics.

[48] Arispe N, Doh M, De Maio A (2002). Lipid interaction differentiates the constitutive and stress-induced heat shock proteins Hsc70 and Hsp70. Cell Stress Chaperones.

[49] Johnson M (2008). NCBI BLAST: a better web interface. Nucleic acids research.

[50] Tamura K, Stecher G, Peterson D, Filipski A, Kumar S (2013). MEGA6: Molecular Evolutionary Genetics Analysis version 6.0. Mol Biol Evol.

[51] Beaumont MA, Nichols RA (1996). Evaluating loci for use in the genetic analysis of population structure. P Roy Soc B-Biol Sci.

[52] Foll M, Gaggiotti O (2008). A Genome-Scan Method to Identify Selected Loci Appropriate for Both Dominant and Codominant Markers: A Bayesian Perspective. Genetics.

[53] Antao, T., Lopes, A., Lopes, R. J., Beja-Pereira, A. & Luikart, G. LOSITAN: A workbench to detect molecular adaptation based on a F(st)-outlier method. *Bmc Bioinformatic*s 9, 10.1186/1471-2105-9-323 (2008). 10.1186/1471-2105-9-323 PMC2515854 18662398

[54] Li H (2011). Tabix: fast retrieval of sequence features from generic TAB-delimited files. Bioinformatics.

[55] Lischer HEL, Excoffier L (2012). PGDSpider: an automated data conversion tool for connecting population genetics and genomics programs. Bioinformatics.

[56] Hecht, M., Bromberg, Y. & Rost, B. Better prediction of functional effects for sequence variants. *Bmc Genomic*s **16**, 10.1186/1471-2164-16-S8-S1 (2015). 10.1186/1471-2164-16-S8-S1 PMC4480835 26110438

[57] Flanagan SE, Patch AM, Ellard S (2010). Using SIFT and PolyPhen to Predict Loss-of-Function and Gain-of-Function Mutations. Genet Test Mol Bioma.

[58] McCallister C, Kdeiss B, Nikolaidis N (2015). Biochemical characterization of the interaction between HspA1A and phospholipids. Cell Stress Chaperones.

[59] Collins TJ (2007). ImageJ for microscopy. Biotechniques.

[60] Burgess A (2010). Loss of human Greatwall results in G2 arrest and multiple mitotic defects due to deregulation of the cyclin B-Cdc2/PP2A balance. Proceedings of the National Academy of Sciences of the United States of America.

[61] Zhuravleva A, Clerico EM, Gierasch LM (2012). An interdomain energetic tug-of-war creates the allosterically active state in Hsp70 molecular chaperones. Cell.

