# Supplementary material for: Concurrent action of purifying selection and gene conversion results in extreme conservation of the major stress-inducible Hsp70 genes in mammals
Source: Sci Rep. 2018 Mar 23;8:5082. doi: 10.1038/s41598-018-23508-x (PMC5865164; doi:10.1038/s41598-018-23508-x)
Supplement: Supplementary file 1 — Supplementary Material [file 41598_2018_23508_MOESM1_ESM.pdf]

Concurrent action of purifying selection and gene conversion results in extreme conservation of the major stress-inducible Hsp70 genes in mammals

Kyle Hess, Ryan Oliverio, Peter Nguyen, Dat Le, Jacqueline Ellis, Brianna Kdeiss, Sara Ord, Dimitra Chalkia, Nikolas Nikolaidis

Supplementary Material Online

Supplementary Tables S1-S10 (pages 2-16)

Supplementary Figs S1-S8 (pages 17-35)

Supplementary Table S1: Sequence comparisons between several HspA1 cluster genes show that the amino acid identity is considerably higher than the nucleotide identity.
Nucleotide and amino acid pairwise identities were calculated with MEGA 6.0. Nucleotide identities are showed above the diagonal and amino acid identities below the diagonal

		1	2	3	4	5	6	7	8	9	10	11	12	13	14	15	16	17	18	19	20	21	22	23	24	25	26
1	human HSPA1A NP_005336		99.74	81.16	91.58	91.68	82.21	92.36	92.36	82.78	94.77	94.77	79.96	98.06	98.06	80.85	92.15	91.68	84.77	94.14	94.56	78.55	78.55	80.38	93.46	93.35	80.64
2	human HSPA1B NP_005337	100.00	-	81.11	91.52	91.63	82.10	92.31	92.31	82.57	94.82	94.82	79.85	98.01	98.01	80.80	92.20	91.73	84.62	94.19	94.61	78.49	78.44	80.22	93.46	93.35	80.59
3	human HSPA1L NP_005518	89.95	89.95	-	78.44	78.39	85.30	78.44	78.44	84.56	80.06	80.06	90.01	81.06	81.16	95.03	77.76	78.49	84.41	79.49	80.01	88.64	81.95	80.80	80.95	81.06	90.21
4	mouse HSPA1A NP_034609	95.45	95.45	88.23	-	99.63	82.68	97.38	97.38	83.36	91.42	91.42	77.55	91.52	91.63	78.44	95.81	95.24	84.77	91.05	91.52	76.35	76.77	78.55	90.42	90.48	78.49
5	mouse HSPA1B NP_034608	95.45	95.45	88.23	100.00	-	82.37	97.59	97.59	83.31	91.58	91.58	77.39	91.63	91.73	78.39	95.92	95.24	84.72	91.21	91.68	76.24	76.61	78.44	90.53	90.58	78.55
6	mouse HSPA1L NP_038586	89.48	89.48	94.98	88.38	88.38	-	82.63	82.63	94.56	81.74	81.74	85.77	82.21	82.10	85.45	81.21	82.21	91.47	81.16	81.79	84.30	78.65	78.34	81.74	81.89	86.55
7	rat HSPA1A NP_114177	96.70	96.70	89.17	98.43	98.43	89.17	-	100.00	84.09	92.46	92.46	77.60	92.41	92.52	78.28	96.18	95.29	85.24	92.05	92.52	76.24	76.82	78.49	90.84	90.69	78.44
8	rat HSPA1B NP_997669	96.70	96.70	89.17	98.43	98.43	89.17	100.00	-	84.09	92.46	92.46	77.60	92.41	92.52	78.28	96.18	95.29	85.24	92.05	92.52	76.24	76.82	78.49	90.84	90.69	78.44
9	rat HSPA1L NP_997711	89.32	89.32	94.82	88.54	88.54	99.22	89.32	89.32	-	83.05	83.05	84.67	82.99	82.99	84.67	82.47	83.36	92.41	82.31	82.84	83.10	78.49	78.44	82.31	82.37	85.82
10	cattle HSPA1A DAA16342	98.90	98.90	89.80	95.76	95.76	89.80	96.86	96.86	89.64	-	100.00	79.49	94.71	94.71	79.85	92.05	91.47	84.72	97.91	98.01	78.18	77.92	79.54	93.04	92.83	79.64
11	cattle HSPA1B DAA16344	98.90	98.90	89.80	95.76	95.76	89.80	96.86	96.86	89.64	100.00	-	79.49	94.71	94.71	79.85	92.05	91.47	84.72	97.91	98.01	78.18	77.92	79.54	93.04	92.83	79.64
12	cattle HSPA1L DAA16392	90.11	90.11	96.23	88.38	88.38	96.08	89.17	89.17	95.60	89.95	89.95	-	80.06	80.06	89.80	76.98	77.81	84.62	78.81	79.38	96.39	81.42	80.48	80.43	80.64	90.16
13	monkey (Aotus) HSPA1A XP_012292128	99.69	99.69	89.80	95.13	95.13	89.64	96.39	96.39	89.48	98.59	98.59	90.27	-	99.90	80.95	91.89	91.42	84.46	94.24	94.66	78.70	78.86	80.85	93.46	93.35	80.85
14	monkey (Aotus) HSPA1B XP_012292127	99.69	99.69	89.80	95.13	95.13	89.64	96.39	96.39	89.48	98.59	98.59	90.27	100.00	-	81.06	91.89	91.42	84.35	94.24	94.66	78.70	78.86	80.85	93.56	93.46	80.85
15	monkey (Aotus) HSPA1L XP_012292126	89.48	89.48	95.60	87.13	87.13	94.19	88.54	88.54	94.03	89.32	89.32	95.29	89.64	89.64	-	77.50	78.23	84.62	79.38	79.85	88.23	81.58	80.59	81.06	81.00	89.95
16	hamster HSPA1A XP_003508521	95.60	95.60	88.23	96.86	96.86	88.23	97.33	97.33	88.38	95.76	95.76	88.23	95.60	95.60	87.44	-	98.80	86.29	91.78	92.10	75.93	76.50	77.81	90.53	90.32	77.71
17	hamster HSPA1B XP_003508520	94.98	94.98	89.17	96.55	96.55	89.48	96.70	96.70	89.64	95.13	95.13	89.17	94.98	94.98	88.07	98.74	-	87.34	91.31	91.63	76.66	76.66	77.86	89.90	89.69	78.23
18	hamster HSPA1L XP_007647281	89.64	89.64	94.82	88.23	88.23	98.74	89.01	89.01	98.59	89.95	89.95	95.60	89.80	89.80	94.03	88.07	89.01	-	84.20	84.51	83.20	78.39	78.07	83.41	83.36	85.35
19	goat HSPA1A NP_001272632	97.65	97.65	88.70	94.82	94.82	88.70	95.92	95.92	88.54	98.74	98.74	88.85	97.33	97.33	88.23	94.82	94.19	88.85	-	98.95	77.55	77.45	79.28	92.41	92.15	78.91
20	goat HSPA1B XP_013829481	98.74	98.74	89.80	96.08	96.08	89.64	97.17	97.17	89.48	99.69	99.69	90.11	98.43	98.43	89.32	96.08	95.45	89.80	98.74	-	78.02	77.97	79.75	92.67	92.52	79.54
21	goat HSPA1L NP_001301135	86.50	86.50	92.15	84.93	84.93	91.84	85.56	85.56	91.37	86.34	86.34	95.45	86.66	86.66	91.21	84.77	85.56	91.37	85.40	86.50	-	80.06	79.38	79.23	79.43	88.75
22	opossum HSPA1A/B XP_001368952	94.66	94.66	90.27	92.31	92.31	89.95	93.09	93.09	90.27	94.03	94.03	90.58	94.82	94.82	89.32	92.46	92.46	90.42	92.94	94.19	87.13	-	92.10	79.70	79.54	81.37
23	opossum HSPA1L XP_001368926	92.31	92.31	92.31	90.11	90.11	91.68	91.21	91.21	91.84	92.15	92.15	92.15	92.46	92.46	91.84	90.58	90.74	91.84	91.05	92.15	88.38	93.09	-	81.53	81.32	80.69
24	elephant HSPA1A ENSLAFG00000017019	98.27	98.27	90.11	95.45	95.45	89.95	96.70	96.70	89.80	98.12	98.12	90.74	97.96	97.96	89.48	95.76	95.13	90.11	97.17	98.43	87.13	94.35	91.68	-	99.37	81.16
25	elephant HSPA1B ENSLAFP00000022914	98.27	98.27	90.11	95.29	95.29	89.95	96.39	96.39	89.80	97.96	97.96	90.74	97.96	97.96	89.32	95.45	94.82	90.11	96.86	98.12	87.13	94.35	91.68	99.37	-	81.58
26	elephant HSPA1L ENSLAFP00000023060	90.11	90.11	96.55	89.01	89.01	95.29	89.64	89.64	95.60	90.27	90.27	96.86	90.27	90.27	95.13	88.85	89.80	95.76	89.17	90.42	92.94	91.21	92.62	90.74	90.89	-

Supplementary Table S2: Synonymous (ps; below diagonal) and non-synonymous (pn; above diagonal) distances (and their standard errors in blue font color) computed using the modified Nei-Gojobori method between several HspA1 cluster genes show that the ps values are considerably higher than the pn ones

		1		2		3		4		5		6		7		8		9		10		11		12		13		14		15		16		17		18		19		20		21		22		23		24		25		26	
1	human HSPA1A NM_005345			0.00	0.00	0.06	0.01	0.03	0.00	0.03	0.00	0.07	0.01	0.02	0.00	0.02	0.00	0.07	0.01	0.01	0.00	0.01	0.00	0.07	0.01	0.00	0.00	0.00	0.00	0.07	0.01	0.03	0.01	0.03	0.01	0.07	0.01	0.01	0.00	0.01	0.00	0.09	0.01	0.06	0.01	0.04	0.01	0.01	0.00	0.01	0.00	0.06	0.01
2	human HSPA1B NM_005346	0.01	0.00			0.06	0.01	0.03	0.00	0.03	0.00	0.07	0.01	0.02	0.00	0.02	0.00	0.07	0.01	0.01	0.00	0.01	0.00	0.07	0.01	0.00	0.00	0.00	0.00	0.07	0.01	0.03	0.01	0.03	0.01	0.07	0.01	0.01	0.00	0.01	0.00	0.09	0.01	0.06	0.01	0.04	0.01	0.01	0.00	0.01	0.00	0.06	0.01
3	human HSPA1L NM_005527	0.48	0.02	0.49	0.02			0.08	0.01	0.08	0.01	0.03	0.01	0.07	0.01	0.07	0.01	0.03	0.00	0.07	0.01	0.07	0.01	0.02	0.00	0.07	0.01	0.07	0.01	0.02	0.00	0.08	0.01	0.08	0.01	0.03	0.01	0.07	0.01	0.07	0.01	0.04	0.01	0.05	0.01	0.07	0.01	0.07	0.01	0.07	0.01	0.02	0.00
4	mouse HSPA1A NM_010479	0.22	0.02	0.23	0.02	0.54	0.02			0.00	0.00	0.08	0.01	0.01	0.00	0.01	0.00	0.08	0.01	0.02	0.00	0.02	0.00	0.08	0.01	0.03	0.01	0.03	0.01	0.09	0.01	0.02	0.00	0.02	0.00	0.08	0.01	0.03	0.00	0.02	0.00	0.10	0.01	0.07	0.01	0.05	0.01	0.02	0.00	0.02	0.00	0.07	0.01
5	mouse HSPA1B NM_010478	0.22	0.02	0.22	0.02	0.54	0.02	0.01	0.00			0.08	0.01	0.01	0.00	0.01	0.00	0.08	0.01	0.02	0.00	0.02	0.00	0.08	0.01	0.03	0.01	0.03	0.01	0.09	0.01	0.02	0.00	0.02	0.00	0.08	0.01	0.03	0.00	0.02	0.00	0.10	0.01	0.07	0.01	0.05	0.01	0.02	0.00	0.02	0.00	0.07	0.01
6	mouse HSPA1L NM_013558	0.43	0.02	0.43	0.02	0.43	0.02	0.40	0.02	0.41	0.02			0.08	0.01	0.08	0.01	0.00	0.00	0.07	0.01	0.07	0.01	0.03	0.00	0.07	0.01	0.07	0.01	0.04	0.01	0.09	0.01	0.08	0.01	0.01	0.00	0.08	0.01	0.07	0.01	0.05	0.01	0.06	0.01	0.07	0.01	0.07	0.01	0.07	0.01	0.03	0.00
7	rat HSPA1A NM_031971	0.21	0.02	0.21	0.02	0.55	0.02	0.07	0.01	0.06	0.01	0.41	0.02			0.00	0.00	0.07	0.01	0.02	0.00	0.02	0.00	0.07	0.01	0.02	0.00	0.02	0.00	0.08	0.01	0.02	0.00	0.02	0.00	0.08	0.01	0.02	0.00	0.02	0.00	0.09	0.01	0.06	0.01	0.05	0.01	0.02	0.00	0.02	0.00	0.07	0.01
8	rat HSPA1B NM_212504	0.21	0.02	0.21	0.02	0.55	0.02	0.07	0.01	0.06	0.01	0.41	0.02	0.00	0.00			0.07	0.01	0.02	0.00	0.02	0.00	0.07	0.01	0.02	0.00	0.02	0.00	0.08	0.01	0.02	0.00	0.02	0.00	0.08	0.01	0.02	0.00	0.02	0.00	0.09	0.01	0.06	0.01	0.05	0.01	0.02	0.00	0.02	0.00	0.07	0.01
9	rat HSPA1L NM_212546	0.41	0.02	0.42	0.02	0.46	0.02	0.38	0.02	0.38	0.02	0.18	0.02	0.36	0.02	0.36	0.02			0.07	0.01	0.07	0.01	0.03	0.00	0.07	0.01	0.07	0.01	0.04	0.01	0.08	0.01	0.07	0.01	0.01	0.00	0.08	0.01	0.07	0.01	0.05	0.01	0.06	0.01	0.07	0.01	0.07	0.01	0.07	0.01	0.03	0.00
10	cattle HSPA1A GJ062795	0.16	0.01	0.16	0.01	0.52	0.02	0.24	0.02	0.23	0.02	0.45	0.02	0.21	0.02	0.21	0.02	0.40	0.02			0.00	0.00	0.07	0.01	0.01	0.00	0.01	0.00	0.07	0.01	0.02	0.00	0.03	0.00	0.07	0.01	0.01	0.00	0.00	0.00	0.09	0.01	0.06	0.01	0.04	0.01	0.01	0.00	0.01	0.00	0.06	0.01
11	cattle HSPA1B GJ062795	0.16	0.01	0.16	0.01	0.52	0.02	0.24	0.02	0.23	0.02	0.45	0.02	0.21	0.02	0.21	0.02	0.40	0.02	0.00	0.00			0.07	0.01	0.01	0.00	0.01	0.00	0.07	0.01	0.02	0.00	0.03	0.00	0.07	0.01	0.01	0.00	0.00	0.00	0.09	0.01	0.06	0.01	0.04	0.01	0.01	0.00	0.01	0.00	0.06	0.01
12	cattle HSPA1L GJ062795	0.52	0.02	0.53	0.02	0.29	0.02	0.57	0.02	0.58	0.02	0.43	0.02	0.58	0.02	0.58	0.02	0.46	0.02	0.53	0.02	0.53	0.02			0.07	0.01	0.07	0.01	0.03	0.01	0.08	0.01	0.08	0.01	0.03	0.00	0.07	0.01	0.07	0.01	0.02	0.00	0.06	0.01	0.07	0.01	0.07	0.01	0.06	0.01	0.02	0.00
13	monkey (Aotus) HSPA1A XM_012436705	0.06	0.01	0.06	0.01	0.49	0.02	0.22	0.02	0.22	0.02	0.43	0.02	0.20	0.02	0.20	0.02	0.40	0.02	0.16	0.02	0.16	0.02	0.52	0.02			0.00	0.00	0.07	0.01	0.03	0.01	0.03	0.01	0.07	0.01	0.02	0.00	0.01	0.00	0.09	0.01	0.06	0.01	0.04	0.01	0.01	0.00	0.01	0.00	0.06	0.01
14	monkey (Aotus) HSPA1B XM_012436704	0.06	0.01	0.06	0.01	0.48	0.02	0.22	0.02	0.21	0.02	0.44	0.02	0.20	0.02	0.20	0.02	0.40	0.02	0.16	0.02	0.16	0.02	0.52	0.02	0.00	0.00			0.07	0.01	0.03	0.01	0.03	0.01	0.07	0.01	0.02	0.00	0.01	0.00	0.09	0.01	0.06	0.01	0.04	0.01	0.01	0.00	0.01	0.00	0.06	0.01
15	monkey (Aotus) HSPA1L XM_012436703	0.48	0.02	0.49	0.02	0.12	0.01	0.53	0.02	0.53	0.02	0.41	0.02	0.55	0.02	0.55	0.02	0.44	0.02	0.51	0.02	0.51	0.02	0.29	0.02	0.48	0.02	0.48	0.02			0.09	0.01	0.08	0.01	0.03	0.01	0.08	0.01	0.07	0.01	0.05	0.01	0.06	0.01	0.07	0.01	0.07	0.01	0.07	0.01	0.03	0.00
16	hamster HSPA1A XM_003508473	0.20	0.02	0.20	0.02	0.56	0.02	0.10	0.01	0.10	0.01	0.43	0.02	0.09	0.01	0.09	0.01	0.40	0.02	0.21	0.02	0.21	0.02	0.58	0.02	0.21	0.02	0.21	0.02	0.55	0.02			0.01	0.00	0.08	0.01	0.03	0.00	0.02	0.00	0.10	0.01	0.07	0.01	0.05	0.01	0.02	0.00	0.03	0.00	0.08	0.01
17	hamster HSPA1B XM_003508472	0.21	0.02	0.21	0.02	0.55	0.02	0.11	0.01	0.11	0.01	0.42	0.02	0.11	0.02	0.11	0.02	0.39	0.02	0.22	0.02	0.22	0.02	0.57	0.02	0.21	0.02	0.21	0.02	0.54	0.02	0.02	0.01			0.08	0.01	0.03	0.00	0.02	0.00	0.10	0.01	0.07	0.01	0.05	0.01	0.03	0.00	0.03	0.00	0.07	0.01
18	hamster HSPA1L XM_007649091	0.34	0.02	0.35	0.02	0.46	0.02	0.32	0.02	0.32	0.02	0.28	0.02	0.31	0.02	0.31	0.02	0.24	0.02	0.35	0.02	0.35	0.02	0.47	0.02	0.36	0.02	0.36	0.02	0.44	0.02	0.26	0.02	0.24	0.02			0.08	0.01	0.07	0.01	0.04	0.01	0.06	0.01	0.07	0.01	0.07	0.01	0.07	0.01	0.02	0.00
19	goat HSPA1A NM_001285703	0.16	0.02	0.16	0.02	0.52	0.02	0.24	0.02	0.23	0.02	0.45	0.02	0.21	0.02	0.21	0.02	0.42	0.02	0.05	0.01	0.05	0.01	0.54	0.02	0.16	0.02	0.16	0.02	0.52	0.02	0.21	0.02	0.22	0.02	0.35	0.02			0.01	0.00	0.09	0.01	0.06	0.01	0.05	0.01	0.01	0.00	0.02	0.00	0.07	0.01
20	goat HSPA1B XM_013974027	0.17	0.02	0.16	0.01	0.52	0.02	0.24	0.02	0.23	0.02	0.45	0.02	0.21	0.02	0.21	0.02	0.41	0.02	0.06	0.01	0.06	0.01	0.54	0.02	0.16	0.02	0.16	0.02	0.51	0.02	0.21	0.02	0.22	0.02	0.36	0.02	0.02	0.01			0.09	0.01	0.06	0.01	0.04	0.01	0.01	0.00	0.01	0.00	0.06	0.01
21	goat HSPA1L NM_001314206	0.52	0.02	0.53	0.02	0.29	0.02	0.57	0.02	0.57	0.02	0.43	0.02	0.58	0.02	0.58	0.02	0.47	0.02	0.53	0.02	0.53	0.02	0.07	0.01	0.52	0.02	0.52	0.02	0.29	0.02	0.58	0.02	0.57	0.02	0.47	0.02	0.54	0.02	0.54	0.02			0.08	0.01	0.08	0.01	0.09	0.01	0.08	0.01	0.04	0.01
22	opossum HSPA1A/B XM_001368889	0.60	0.02	0.60	0.02	0.50	0.02	0.63	0.02	0.63	0.02	0.59	0.02	0.64	0.02	0.64	0.02	0.60	0.02	0.62	0.02	0.62	0.02	0.50	0.02	0.59	0.02	0.59	0.02	0.50	0.02	0.63	0.02	0.63	0.02	0.61	0.02	0.62	0.02	0.62	0.02	0.50	0.02			0.04	0.01	0.06	0.01	0.06	0.01	0.05	0.01
23	opossum HSPA1L XM_001368915	0.57	0.02	0.57	0.02	0.50	0.02	0.60	0.02	0.60	0.02	0.58	0.02	0.61	0.02	0.61	0.02	0.58	0.02	0.59	0.02	0.59	0.02	0.51	0.02	0.55	0.02	0.55	0.02	0.49	0.02	0.62	0.02	0.62	0.02	0.59	0.02	0.58	0.02	0.58	0.02	0.51	0.02	0.16	0.02			0.04	0.01	0.04	0.01	0.06	0.01
24	elephant HSPA1A ENSLAFG00000017019	0.20	0.02	0.20	0.02	0.48	0.02	0.27	0.02	0.26	0.02	0.45	0.02	0.26	0.02	0.26	0.02	0.43	0.02	0.21	0.02	0.21	0.02	0.51	0.02	0.19	0.02	0.19	0.02	0.47	0.02	0.26	0.02	0.28	0.02	0.39	0.02	0.22	0.02	0.23	0.02	0.50	0.02	0.55	0.02	0.53	0.02			0.00	0.00	0.07	0.01
25	elephant HSPA1B ENSLAFP00000022914	0.20	0.02	0.20	0.02	0.48	0.02	0.26	0.02	0.26	0.02	0.45	0.02	0.26	0.02	0.26	0.02	0.43	0.02	0.21	0.02	0.21	0.02	0.51	0.02	0.19	0.02	0.19	0.02	0.47	0.02	0.26	0.02	0.28	0.02	0.40	0.02	0.22	0.02	0.23	0.02	0.50	0.02	0.55	0.02	0.53	0.02	0.01	0.00			0.06	0.01
26	elephant HSPA1L ENSLAFP00000023060	0.50	0.02	0.51	0.02	0.29	0.02	0.55	0.02	0.55	0.02	0.40	0.02	0.56	0.02	0.56	0.02	0.43	0.02	0.54	0.02	0.54	0.02	0.30	0.02	0.50	0.02	0.50	0.02	0.28	0.02	0.57	0.02	0.57	0.02	0.45	0.02	0.55	0.02	0.54	0.02	0.30	0.02	0.51	0.02	0.51	0.02	0.48	0.02	0.47	0.02		

Supplementary Table S3: Polymorphic sites and density for the three HSPA1 and five surrounding genes and their coding sites separately. The data derived from the 1000 Genomes (1000g) and ExAC datasets

		SNP sites (1000g)		SNP sites (ExAC)			coding sites (1000g)		coding sites (ExAC)		
Gene	Region Length (bp)	N	Density[#]	N	Density	SNP sites Density Ratio (ExAC/1000g)	N	Density[#]	N	Density	Coding sites Density Ratio (ExAC/1000g)
HSPA1A	2432	39	0.016	46	0.019	1.2	16	0.007	26	0.011	1.6
HSPA1B	2519	46	0.018	83	0.033	1.8	17	0.007	26	0.01	1.5
HSPA1L	6041	153	0.025	307	0.051	2	59	0.01	288	0.048	4.9
VARS	18434	410	0.022	961	0.052	2.3	91	0.005	593	0.032	6.5
LSM2	9587	228	0.024	114	0.012	0.5	2	0	19	0.002	9.5
C6orf48	5155	160	0.031	223	0.043	1.4	10	0.002	48	0.009	4.8
NEU	5246	85	0.016	285	0.054	3.4	24	0.005	178	0.034	7.4
SLC44A4	15853	422	0.027	647	0.041	1.5	61	0.004	337	0.021	5.5

Supplementary Table S4: The distribution of SNP type is significantly different between the HSPA1 and their neighboring genes. This analysis was performed using the ExAC dataset. The same trends are observed when we compared the aggregated SNP distribution of: HSPA1A-1B to all six other genes and HSPA1 (cluster) to all five non-Hsp70 genes for both 1000 Genomes and ExAC datasets

Count / Row %	3' UTR	5' UTR	CDS_other	intron	missense	synonymous	Total
HSPA1A	4	21	4	0	14	12	55
	7.27	38.18	7.27	0	25.45	21.82	
HSPA1B	10	58	2	0	12	14	96
	10.42	60.42	2.08	0	12.5	14.58	
HSPA1L	5	3	27	5	204	84	328
	1.52	0.91	8.23	1.52	62.2	25.61	
C6orf48	14	142	21	22	34	14	247
	5.67	57.49	8.5	8.91	13.77	5.67	
LSM2	11	18	16	68	10	9	132
	8.33	13.64	12.12	51.52	7.58	6.82	
NEU	4	16	77	5	125	53	280
	1.43	5.71	27.5	1.79	44.64	18.93	
SLC44A4	7	7	102	238	251	86	691
	1.01	1.01	14.76	34.44	36.32	12.45	
VARS	6	2	209	206	390	203	1016
	0.59	0.2	20.57	20.28	38.39	19.98	
Total	61	267	458	544	1040	475	2845

Test	ChiSquare	Prob>ChiSq
Likelihood Ratio	1461.325	<.0001*
Pearson	1766.075	<.0001*

1000 Genomes data Distribution of SNP type by gene

In this analysis only HSPA1A and 1B genes were grouped together and compared to the rest sixe genes

Count / Row %	3'URT	5'UTR	CDS_other	intron	missense	synonymous	Total
Six surrounding genes	49	60	28	1075	156	91	1459
	3.36	4.11	1.92	73.68	10.69	6.24	
HSPA1A and HSPA1B	18	34	0	0	12	21	85
	21.18	40	0	0	14.12	24.71	
Total	67	94	28	1075	168	112	1544

Test	ChiSquare	Prob>ChiSq
Likelihood Ratio	262.585	<.0001*
Pearson	331.576	<.0001*

1000 Genomes data Distribution of SNP type by gene

In this analysis all three HSPA1 genes were grouped together and compared to the rest five genes

Count / Row %	3'UTR	5'UTR	CDS_other	intron	missense	synonymous	Total
HSPA1	24	60	2	60	49	43	238
	10.08	25.21	0.84	25.21	20.59	18.07	
surrounding	43	34	26	1015	119	69	1306
	3.29	2.6	1.99	77.72	9.11	5.28	
Total	67	94	28	1075	168	112	1544

Test	ChiSquare	Prob>ChiSq
Likelihood Ratio	287.597	<.0001*
Pearson	341.304	<.0001*

ExAC data Distribution of SNP type by gene

In this analysis all 3 HSPA1 genes were grouped together and compared to the rest five genes

Count / Row %	3' UTR	5' UTR	CDS_other	intron	missense	synonymous	Total
HSPA1-cluster	19	82	33	5	230	110	479
	3.97	17.12	6.89	1.04	48.02	22.96	
non-cluster	42	185	425	539	810	365	2366
	1.78	7.82	17.96	22.78	34.23	15.43	
Total	61	267	458	544	1040	475	2845

Test	ChiSquare	Prob>ChiSq
Likelihood Ratio	267.029	<.0001*
Pearson	208.667	<.0001*

ExAC data Distribution of SNP type by gene

In this analysis only HSPA1A and 1B genes were grouped together and compared to the rest sixe genes

Count / Total % / Col % / Row %	3' UTR	5' UTR	intron	missense	other	synonymous	Total
Six surrounding genes	47	188	544	1014	452	449	2694
	1.65	6.61	19.12	35.64	15.89	15.78	94.69
	77.05	70.41	100	97.5	98.69	94.53	
	1.74	6.98	20.19	37.64	16.78	16.67	
HSPA1A and HSPA1B	14	79	0	26	6	26	151
	0.49	2.78	0	0.91	0.21	0.91	5.31
	22.95	29.59	0	2.5	1.31	5.47	
	9.27	52.32	0	17.22	3.97	17.22	
Total	61	267	544	1040	458	475	2845
	2.14	9.38	19.12	36.56	16.1	16.7	

Test	ChiSquare	Prob>ChiSq
Likelihood Ratio	281.758	<.0001*
Pearson	412.368	<.0001*

Supplementary Table S5: The Minor Allele Frequency (MAF) between the HSPA1 cluster and its surrounding genes is significantly different, yet the effect sizes of the differences were small. This analysis was performed using the ExAC dataset

Count<br>Col %<br>Row %	HSPA1	surrounding	Total
common	9<br>1.88<br>27.27	24<br>1.01<br>72.73	33
low-frequency	13<br>2.71<br>28.89	32<br>1.35<br>71.11	45
rare	457<br>95.41<br>16.52	2310<br>97.63<br>83.48	2767
Total	479	2366	2845

**Test**	**ChiSquare**	**Prob>ChiSq**
Likelihood Ratio	6.467	0.0394*
Pearson	7.438	0.0243*

Supplementary Table S6: SNP frequencies in HSPA1 genes. The data used in the top table were downloaded from 1000 Genomes FTP site via Allele Frequency Calculator tool; the data used in the bottom table were downloaded from the ExAC database

Count / Row %	common	low-frequency	rare	Total
HSPA1A	4	4	29	37
	10.81	10.81	78.38	
HSPA1B	4	11	28	43
	9.3	25.58	65.12	
HSPA1L	10	27	115	152
	6.58	17.76	75.66	
Total	18	42	172	232

Test	ChiSquare	Prob>ChiSq
Likelihood Ratio	3.892	0.4208
Pearson	3.883	0.4221

Count / Row %	common	low-frequency	rare	Total
HSPA1A	4	1	50	55
	7.27	1.82	90.91	
HSPA1B	2	7	87	96
	2.08	7.29	90.63	
HSPA1L	3	5	320	328
	0.91	1.52	97.56	
Total	9	13	457	479

Test	ChiSquare	Prob>ChiSq
Likelihood Ratio	14.699	0.0054*
Pearson	19.952	0.0005*

Supplementary Table S7: Properties of the SNPs selected for functional studies and the primer sequences used for cloning to the pEGFP vector and the site directed mutagenesis (bottom table)

dbSNP rs# clusster id	Variant	Population	AA conservation	BLOSUM 65 (80) scores	PolyPhen	SIFT	SNAP score	AA Class Change
rs200520712	S16P	European American (clinical)	95%	-1(-1)	Probably Damaging	Deleterious	89	# to 0
rs367576856	S16Y	African American	95%	-2(-2)	Probably Damaging	Deleterious	82	# to 0
rs757670500	R36C	European (ExAc)	92%	-4(-4)	Benign	Deleterious	54	+ to #
rs1043620	I74T	Europesn (ExAc)	94%	-1(-1)	Probably Damaging	Deleterious	35	0 to #
rs112626070	I480N	European American (clinical)	95%	-3(-4)	Probably Damaging	Deleterious	88	0 to #
rs200083915	F592S	European American (ExAc)	86%	-2(-3)	Possibly Damaging	Deleterious	30	0 to #

AA class change is based on the charge of the amino acid in a solution at neutral pH. 0 - nonpolar, # - polar,uncharged, + (-) – basic (acidic). † Population as defined by Lek et al., 2016.

**Primer Name**	**Primer Sequence (5-3)** (the restriction site is underlined)
5-HSPA1A-XhoI	CAT<u>CTCGAG</u>CATGGCCAAGAACACGGCG
3-HSPA1A-BamHI	CCG<u>GGATCC</u>ATCCACCTCCTCGATGGT
A1A_S16P_F	CGACCTGGGCACCACCTACCCGTGCGTGGGCGTGTTCCAG
A1A_S16P_R	CTGGAACACGCCCACGCACGGGTAGGTGGTGCCCAGGTCG
A1A_S16Y_F	GACCTGGGCACCACCTACTATTGCGTGGGCGTGTTCCAGC
A1A_S16Y_R	GCTGGAACACGCCCACGCAATAGTAGGTGGTGCCCAGGTC
A1A_R36C_F	CGCCAACGACCAGGGCAACTGCACGACCCCCAGCTACGTG
A1A_R36C_R	CACGTAGCTGGGGGTCGTGCAGTTGCCCTGGTCGTTGGCG
A1A_I74T_F	GTTCGACGCGAAGCGGCTGACCGGCCGCAAGTTCGGCGATGC
A1A_I74T_R	GCATCGCCGAACTTGCGGCCGGTCAGCCGCTTCGCGTCGAAC
A1A_I480N_F	GATCGAGGTGACCTTCGACAATGACGCCAACGGCATCCTGAAC
A1A_I480N_R	GTTCAGGATGCCGTTGGCGTCATTGTCGAAGGTCACCTCGATC
A1A_F592S_F	GCTGGCCGACAAGGAGGAGAGCGTGCACAAGCGGGAGGAG
A1A_F592S_R	CTCCTCCCGCTTGTGCACGCTCTCCTCCTTGTCGGCCAGC
A1A_K71A_F	GAACACCGTGTTCGACGCGGCGCGGCTGATCGGCCGCCGCAAG
A1A_K71A_R	CTTGCGGCGGCCGATCAGCCGCGCCGCGTCGAACACGGTGTTC

Supplementary Table S8: Binding and thermal results of the Isothermal Titration Calorimetry (ITC) assays using purified recombinant HspA1A proteins (WT and mutated variants) with ATP and ADP. N: reaction stoichiometry; dH: enthalpy; dS: entropy; Kd: dissociation constant; Cell: instrument cell containing protein; Syringe: instrument syringe for ligand titration

Protein (Cell)	Syringe (ligand)	N	Kd (mM)	dH (kJ/mol)	dS (J/mol*k)
WT1	ATP	1.041	2.93E-05	-53.93	-104.66
WT2	ATP	1.001	3.09E-05	-57.76	-107.41
WT3	ATP	1.006	2.59E-05	-60.63	-118.62
K71A_1	ATP	1.016	3.52E-04	-38.38	-62.59
K71A_2	ATP	1.039	2.94E-04	-42.78	-67.22
K71A_3	ATP	1.065	3.57E-04	-41.27	-60.74
S16P_1	ATP	0.957	5.65E-04	-53.46	-104.14
S16P_2	ATP	1.006	2.60E-04	-52.7	-108.11
S16P_3	ATP	0.953	3.11E-04	-54.31	-106.42
S16Y_1	ATP	1.059	2.81E-05	-49.38	-78.49
S16Y_2	ATP	1.008	3.13E-05	-48.93	-77.82
S16Y_3	ATP	1.046	2.70E-05	-51.68	-79.23
R346C_1	ATP	0.991	2.25E-05	-53.73	-97.95
R346C_2	ATP	1.017	3.73E-05	-52.36	-97.91
R346C_3	ATP	0.987	2.67E-05	-52.03	-99.11
I74T_1	ATP	1.037	3.20E-05	-55.86	-86.03
I74T_2	ATP	1.035	4.16E-05	-52.36	-85.87
I74T_3	ATP	1.009	3.72E-05	-50.87	-84.68
I480N_1	ATP	1.028	1.77E-05	-50.51	-78.44
I480N_2	ATP	1.033	2.25E-05	-52.02	-82.57
I480N_3	ATP	1.06	1.75E-05	-52.62	-81.78
F592S_1	ATP	1.031	2.12E-05	-55.09	-95.28
F592S_2	ATP	1.028	2.36E-05	-54.54	-94.36
F592S_3	ATP	1.034	2.14E-05	-53.66	-95.13

Protein (Cell)	Syringe (ligand)	N	Kd (mM)	dH (kJ/mol)	dS (J/mol*k)
WT1	ADP	0.994	7.12E-05	-39.95	-58.67
WT2	ADP	1.024	8.28E-05	-42.71	-65.06
WT3	ADP	0.997	9.85E-05	-42.75	-66.67
K71A_1	ADP	1.039	4.79E-04	-28.74	-32.84
K71A_2	ADP	1.037	4.56E-04	-26.85	-35.61
K71A_3	ADP	1.091	4.82E-04	-30.58	-28.19
S16P_1	ADP	1.004	1.00E-04	-38.25	-55.25
S16P_2	ADP	0.981	1.45E-04	-41.94	-55.92
S16P_3	ADP	0.988	1.88E-04	-39.53	-54.48
S16Y_1	ADP	1.017	5.67E-05	-44.65	-69.46
S16Y_2	ADP	1.02	7.33E-05	-45.18	-72.38
S16Y_3	ADP	1.096	7.55E-05	-46.56	-71.24
R346C_1	ADP	0.984	1.91E-04	-44.15	-43.31
R346C_2	ADP	1.01	1.69E-04	-48.31	-41.43
R346C_3	ADP	1.016	1.16E-04	-47.6	-44.43
I74T_1	ADP	1.043	8.69E-05	-44.28	-70.79
I74T_2	ADP	1.031	9.82E-05	-43.84	-68.18
I74T_3	ADP	1.043	8.17E-05	-44.31	-70.35
I480N_1	ADP	1.09	5.79E-05	-40.95	-49.2
I480N_2	ADP	1.031	6.23E-05	-39.48	-51.9
I480N_3	ADP	1.009	5.87E-05	-41.68	-48.79
F592S_1	ADP	1.043	4.86E-05	-35.82	-31.12
F592S_2	ADP	1.024	5.22E-05	-34.23	-32.81
F592S_3	ADP	1.004	6.13E-05	-33.62	-35.15

Supplementary Table S9: Locitan results on common and low-frequency polymorphisms on HSPA1 genes. Identified outlier loci are highlighted green. P-values deem the results as non significant

Het Expected Heterozygosity.

F_{ST} The proportion of the total genetic variance contained in a population relative to the total genetic variance. Values can range from 0 to 1.

Gene	SNP ID	Het	F_{ST}	F_{ST}/Het	P(Simul F_{ST}<sample F_{ST})
HSPA1A	rs16867582	0.04	0	0.11	0.15
	rs11557922	0.06	0.01	0.17	0.14
	rs506770	0.27	0.05	0.19	0.42
	rs1008438	0.51	0.11	0.22	0.62
	rs4713489	0.09	0.02	0.25	0.24
	rs34416404	0.09	0.02	0.25	0.24
	rs1043618	0.51	0.16	0.31	0.83
	rs562047	0.34	0.16	0.48	0.87
	rs1043620	0.07	0.04	0.58	0.54
	rs34378923	0.02	0.02	0.64	0.5
	rs33934112	0.02	0.02	0.96	0.5
	rs12526722	0.02	0.02	1.12	0.5
	rs34397183	0.01	0.02	1.75	1
HSPA1B	rs56280220	0.03	0	0.11	0.24
	rs13217108	0.04	0	0.11	0.24
	rs6457452	0.2	0.03	0.15	0.39
	rs2763979	0.5	0.11	0.22	0.9
	rs144223778	0.12	0.04	0.31	0.49
	rs41312325	0.07	0.03	0.35	0.47
	rs11576012	0.02	0.01	0.57	0.65
	rs11576013	0.08	0.06	0.8	0.72
	rs34004874	0.01	0.01	0.81	0.81
	rs11576014	0.03	0.03	0.85	0.48
	rs483638	0.05	0.08	1.5	0.87
	rs17201199	0.02	0.03	1.54	0.87
	rs36058616	0.01	0.02	1.6	0.84
	rs7771177	0.01	0.02	1.76	1
	rs34396430	0.01	0.02	1.78	0.85
	rs17207580	0.01	0.02	1.91	1
	rs2607018	0.01	0.02	2.41	0.87
	rs113094932	0	-100		0.5
HSPA1L	rs149831348	0.02	0	0.07	0.02

rs16867582	0.04	0	0.11	0.18
rs2075799	0.25	0.03	0.12	0.27
rs11557922	0.06	0.01	0.17	0.16
rs34636308	0.06	0.01	0.17	0.16
rs34431565	0.06	0.01	0.18	0.25
rs7757496	0.19	0.04	0.18	0.37
rs34814308	0.06	0.01	0.19	0.26
rs2227957	0.02	0	0.21	0.03
rs1008438	0.51	0.11	0.22	0.78
rs2227955	0.09	0.02	0.25	0.31
rs4713489	0.09	0.02	0.25	0.31
rs2075800	0.41	0.1	0.25	0.79
rs34416404	0.09	0.02	0.25	0.31
rs35570187	0.15	0.04	0.26	0.43
rs2227956	0.22	0.06	0.27	0.55
rs1043618	0.51	0.16	0.31	0.94
rs12213612	0.1	0.05	0.48	0.56
rs562047	0.34	0.16	0.48	0.95
rs118191775	0.04	0.02	0.53	0.39
rs481825	0.19	0.1	0.54	0.82
rs1043620	0.07	0.04	0.58	0.59
rs34378923	0.02	0.02	0.64	0.15
rs115333512	0.03	0.02	0.77	0.52
rs33934112	0.02	0.02	0.96	0.48
rs35326839	0.07	0.07	1	0.79
rs12526722	0.02	0.02	1.12	0.34
rs34620296	0.01	0.01	1.21	0.7
rs35347921	0.01	0.01	1.21	0.7
rs34397183	0.01	0.02	1.75	1
rs35804213	0.01	0.02	1.91	1
rs482145	0.01	0.02	1.99	0.72
rs55708070	0.01	0.02	2.07	0.76
rs112798021	0.04	0.08	2.15	1
rs35858615	0.03	0.07	2.15	1
rs114406544	0.01	0.02	2.41	0.76
rs34791928	0.01	0.03	2.41	1
rs34372373	0.01	0.03	2.43	1

Supplementary Table S10: BayeScan results on common and low-frequency polymorphisms on HSPA1 genes. Identified outlier loci are highlighted green. Q-values deem the results as non significant

prob The posterior probability for the model including selection.

log10(PO) The logarithm of Posterior Odds to base 10 for the model including selection. Note that this value is arbitrarily fixed to 1000 when the posterior probability is 1 (should be infinity).

q-value The q-value for the model including selection. The FDR analogue of the p-value (note that a q-value is only defined in the context of multiple testing, whereas a p-value is defined on a single test). The q-value of given locus is the minimum FDR at which this locus may become significant.

alpha The estimated alpha coefficient indicating the strength and direction of selection. A positive value of alpha suggests diversifying selection, whereas negative values suggest balancing or purifying selection.

FST The F_{ST} coefficient averaged over populations. In each population F_{ST} is calculated as the posterior mean using model averaging.

Gene	SNP ID	prob	log_{10}(PO)	q-value	alpha	F_{ST}
HSPA1A	rs1008438	0.087017	-1.0209	0.81928	0.033308	0.09081
	rs1043618	0.052611	-1.2555	0.87233	0.007208	0.086409
	rs1043620	0.073215	-1.1024	0.84197	-0.01821	0.084977
	rs114151859	0.36267	-0.24485	0.63733	-0.57436	0.063316
	rs12526722	0.10042	-0.95222	0.803661	-0.04314	0.084364
	rs16867582	0.10662	-0.92319	0.78448	-0.06892	0.082555
	rs33934112	0.17003	-0.68852	0.75725	-0.18657	0.077047
	rs34378923	0.22525	-0.53651	0.70604	-0.27898	0.073026
	rs34397183	0.084017	-1.0375	0.83137	-0.00691	0.086083
	rs34416404	0.21304	-0.56748	0.73301	-0.25459	0.073989
	rs4713489	0.054011	-1.2434	0.86607	-0.00416	0.085445
	rs506770	0.070614	-1.1193	0.85071	-0.02034	0.084599
	rs562047	0.060212	-1.1933	0.85881	-0.00613	0.085416
HSPA1B	rs113094932	0.12683	-0.83789	0.78058	0.11451	0.16165
	rs11576012	0.11802	-0.87349	0.79326	-0.09469	0.13479
	rs11576013	0.088218	-1.0143	0.83597	-0.04069	0.13796
	rs11576014	0.084017	-1.0375	0.84656	-0.02324	0.14003
	rs13217108	0.36507	-0.24034	0.61772	-0.59017	0.10495
	rs144223778	0.14843	-0.7587	0.74711	-0.14557	0.1295
	rs17201199	0.081216	-1.0536	0.85107	0.016985	0.14486
	rs17207580	0.09862	-0.96094	0.81418	0.037428	0.14815
	rs2607018	0.10562	-0.92777	0.80449	0.07635	0.15475
	rs2763979	0.061412	-1.18422	0.863173	-0.01186	0.13985
	rs34004874	0.079216	-1.0653	0.85517	0.00925	0.14417
	rs34396430	0.096419	-0.9718	0.82231	0.037393	0.14851

	rs36058616	0.085217	-1.0308	0.8416	0.00577	0.14387
	rs41312325	0.15043	-0.75186	0.72099	-0.13864	0.13002
	rs483638	0.076215	-1.0835	0.85898	0.031353	0.14616
	rs56280220	0.39948	-0.17703	0.60052	-0.69785	0.10075
	rs6457452	0.20104	-0.59924	0.67814	-0.22848	0.12312
	rs7771177	0.089618	-1.0068	0.82965	0.007118	0.14389
HSPA1L	rs1008438	0.04921	-1.286	0.8878	0.009948	0.069223
	rs1043618	0.060212	-1.1933	0.88095	0.029033	0.070806
	rs1043620	0.070414	-1.1206	0.87566	-0.0155	0.067886
	rs112798021	0.10822	-0.91594	0.83442	0.086499	0.078313
	rs114406544	0.114423	-0.88871	0.81429	0.081529	0.078593
	rs115333512	0.080816	-1.0559	0.8684	-0.03113	0.067608
	rs11557922	0.14383	-0.77471	0.79976	-0.12244	0.063979
	rs118191775	0.082216	-1.0478	0.8645	-0.01777	0.068217
	rs12213612	0.075015	-1.091	0.87208	-0.01626	0.067903
	rs12526722	0.077616	-1.075	0.87026	-0.01911	0.068212
	rs149831348	0.31626	-0.33484	0.67053	-0.51299	0.053081
	rs16867582	0.34267	-0.28291	0.65733	-0.53679	0.051758
	rs2075799	0.083817	-1.0387	0.86237	-0.04005	0.066581
	rs2075800	0.09762	-0.96585	0.85348	0.059419	0.073663
	rs2227955	0.15103	-0.74983	0.77292	-0.14425	0.06304
	rs2227956	0.053211	-1.2503	0.88446	0.006482	0.068992
	rs2227957	0.19944	-0.60358	0.73545	-0.23475	0.060869
	rs33934112	0.081216	-1.0536	0.86651	-0.00706	0.069236
	rs34372373	0.10962	-0.90968	0.83032	0.075198	0.078153
	rs34378923	0.10102	-0.94934	0.83846	-0.0488	0.066922
	rs34397183	0.095219	-0.97782	0.85581	0.042155	0.073775
	rs34416404	0.15323	-0.74242	0.75771	-0.14062	0.063192
	rs34431565	0.14963	-0.75459	0.78399	-0.14488	0.063016
	rs34620296	0.094819	-0.97984	0.85795	0.002047	0.070382
	rs34636308	0.12623	-0.84025	0.80716	-0.10994	0.064096
	rs34791928	0.11082	-0.90436	0.8257	0.089628	0.07909
	rs34814308	0.11222	-0.89822	0.82041	-0.0836	0.065202
	rs35326839	0.070814	-1.118	0.87392	0.032731	0.071663
	rs35347921	0.089818	-1.0058	0.86013	0.036231	0.073424
	rs35570187	0.065813	-1.1521	0.87744	-0.01373	0.067905
	rs35804213	0.09762	-0.96585	0.85348	0.048768	0.074726
	rs35858615	0.09942	-0.95705	0.84211	0.060681	0.07553
	rs4713489	0.14623	-0.76631	0.79271	-0.14172	0.06318
	rs481825	0.061812	-1.1812	0.87922	-0.00811	0.068144

rs482145	0.09922	-0.95802	0.84537	0.038563	0.073978
rs55708070	0.09842	-0.96192	0.84833	0.038942	0.073956
rs562047	0.05161	-1.2643	0.88615	0.009053	0.069181
rs7757496	0.055011	-1.235	0.88273	-0.00843	0.067987

Supplementary Fig. S1: The HspA1A-HspA1B cluster originated early during the evolution of placental mammals and shows intraspecies phylogenetic clades. The evolutionary history was inferred by using the Maximum Likelihood method based on the JTT matrix-based model. The tree with the highest log likelihood (-4644.3204) is shown. The percentage of trees in which the associated taxa clustered together is shown next to the branches. Initial tree(s) for the heuristic search were obtained by applying the Neighbor-Joining method to a matrix of pairwise distances estimated using a JTT model. A discrete Gamma distribution was used to model evolutionary rate differences among sites (5 categories (+G, parameter = 0.6482)). The rate variation model allowed for some sites to be evolutionarily invariable ([+I], 52.3576% sites). The tree is drawn to scale, with branch lengths measured in the number of substitutions per site. All positions containing gaps and missing data were eliminated. There were a total of 635 positions in the final dataset.

Supplementary Fig. S2: HspA1A and HspA1B genes are identical (or almost) identical at both the nucleotide (first page) and amino acid (second) levels. Pairwise sequence alignments between mammalian HspA1A and HspA1B sequences over sliding windows were performed using SWAAP (v.1.0.3).

Supplementary Fig. S3: The amino acid identity between HspA1A (or 1B) and HspA1L sequences is considerably higher than the nucleotide identity. Pairwise sequence alignments between mammalian HspA1A and HspA1B sequences over sliding windows were performed using SWAAP (v.1.0.3).

Supplementary Fig. S4: ps and pn analysis over sliding windows reveals the action of both purifying selection and gene conversion in during the evolution of the HspA1 gene cluster. Synonymous/non-synonymous substitutions over sliding windows were performed using SWAAP (v.1.0.3) using the modified Nei-Gojobori method. The first page contains comparisons between HspA1A and HspA1B gene sequences, while the second page contains comparisons between HspA1A and HspA1L gene sequences.

Supplementary Fig. S5: The distribution of SNPs is very different between the three HSPA1 genes. Synonymous and non-synonymous SNPs were mapped along the amino acid sequence of HSPA1A, HSPA1B, and HSPA1L using sliding window analysis. The domain organization of Hsp70s (from XXX) is depicted on the top. The figures on the left column contain data from 1000 Genomes and the figures on the right from the ExAC databases. The scale between the two columns is different.

Supplementary Fig. S6: Thermal Shift Assay data fitted using the Sigmoidal Boltzmann equation are presented for three protein batches.

Supplementary Fig. S7: Representative Isothermal Titration Calorimetry (ITC) assays using purified recombinant HspA1A proteins (WT and mutated variants) with ATP and ADP. The top graphs are the ITC raw data for 20 automatic injections of nucleotide into the sample cell containing either WT or mutated HspA1A. The bottom graphs represent

ITC binding curves obtained for the interaction between the nucleotide and HspA1A. The data shown are representative of three independent experiments.

Supplementary Fig. S8: Representative images of the intracellular localization of WT and mutated HspA1A variants at 37 ^{o}C and immediately after a 1 hour 42 ^{o}C heat stress assessed by co-localization analysis using organelle specific markers: nucleus (page 1); mitochondria (page 2); lysosomes (page 3); and plasma membrane (page 4). PDM refers to Product of the Differences from the Mean, with images representing only areas where positive PDM values were calculated. Scale bars represent 20μm.

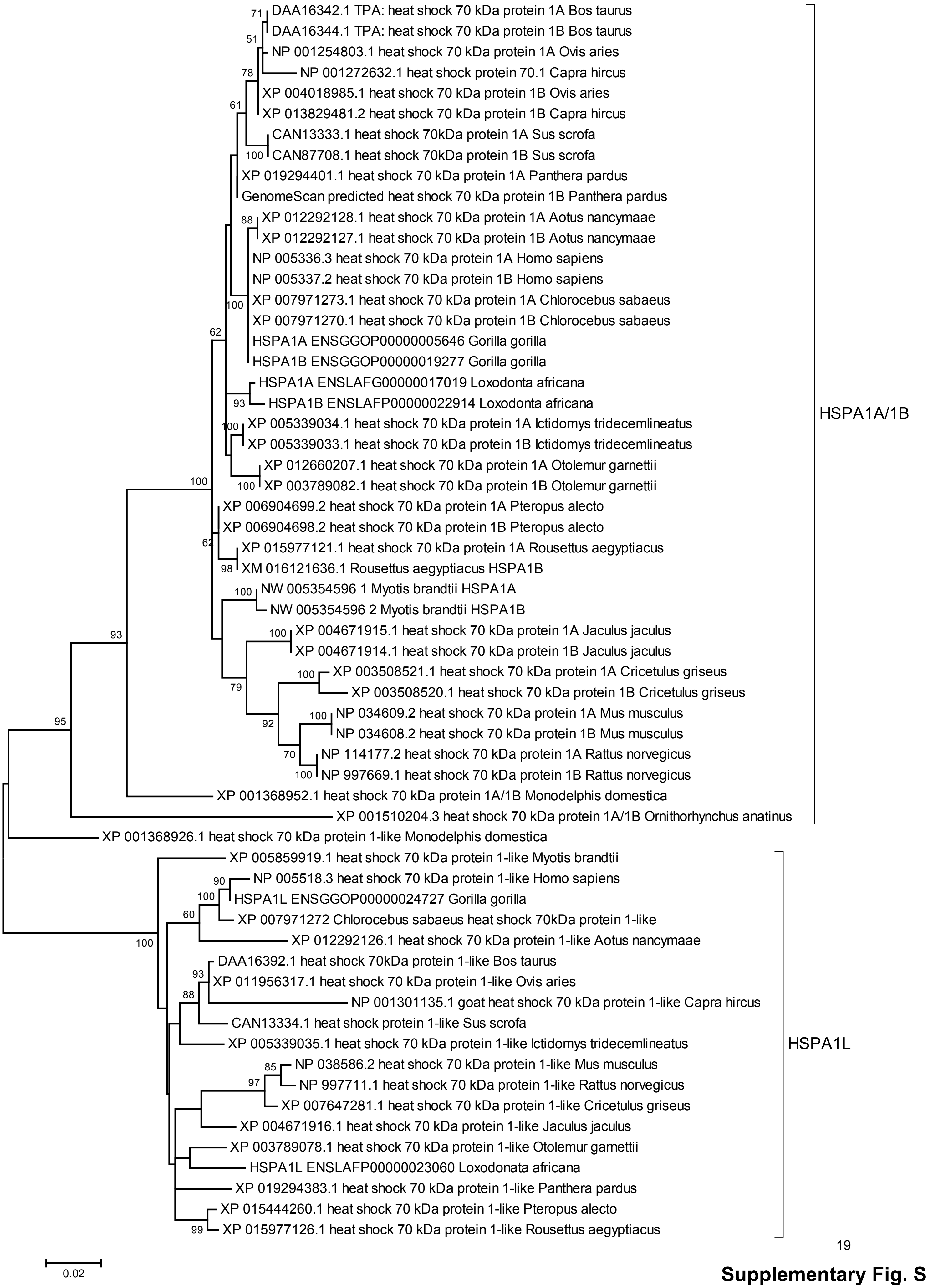

Supplementary Fig. S1

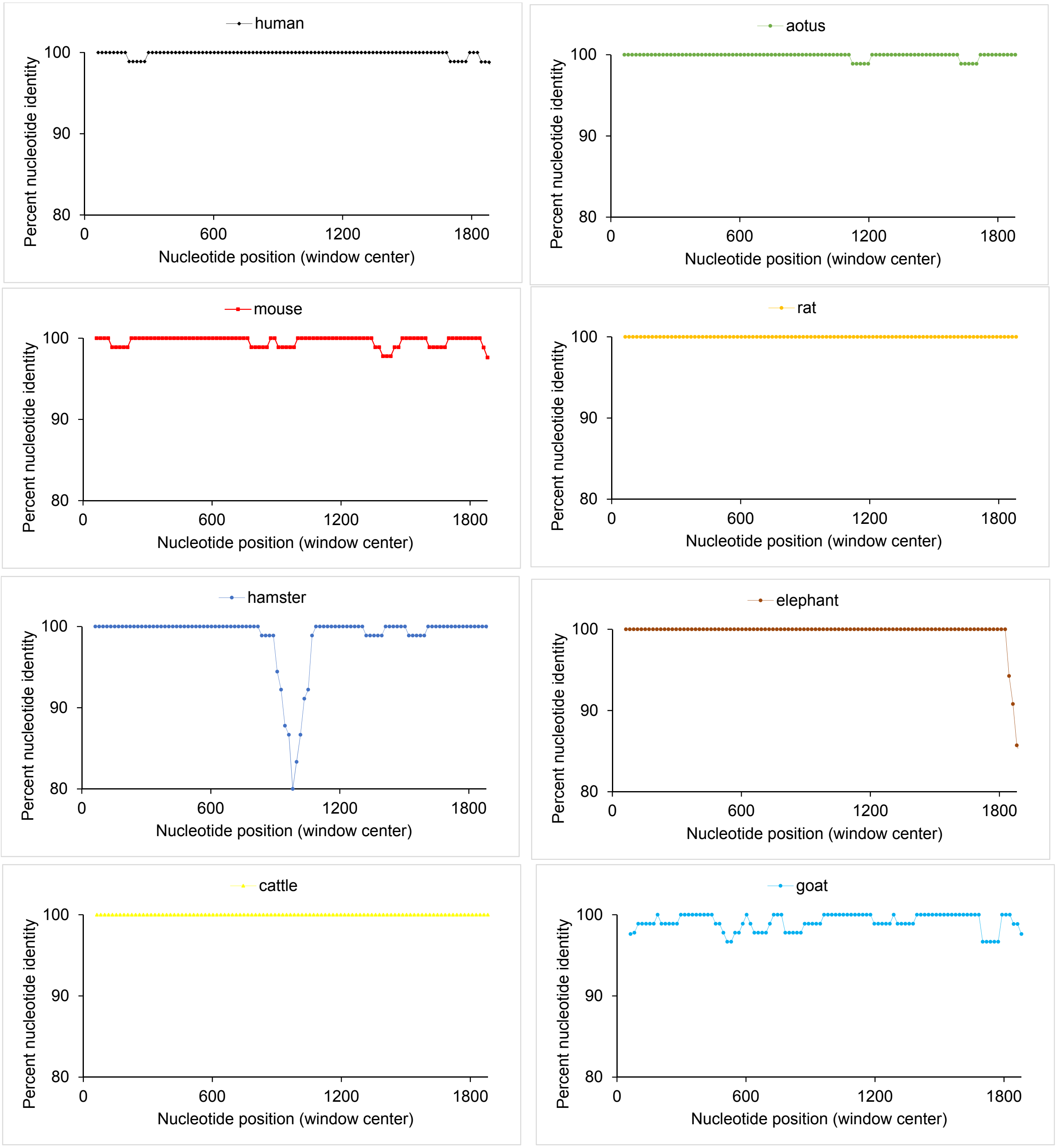

Supplementary Fig. S2

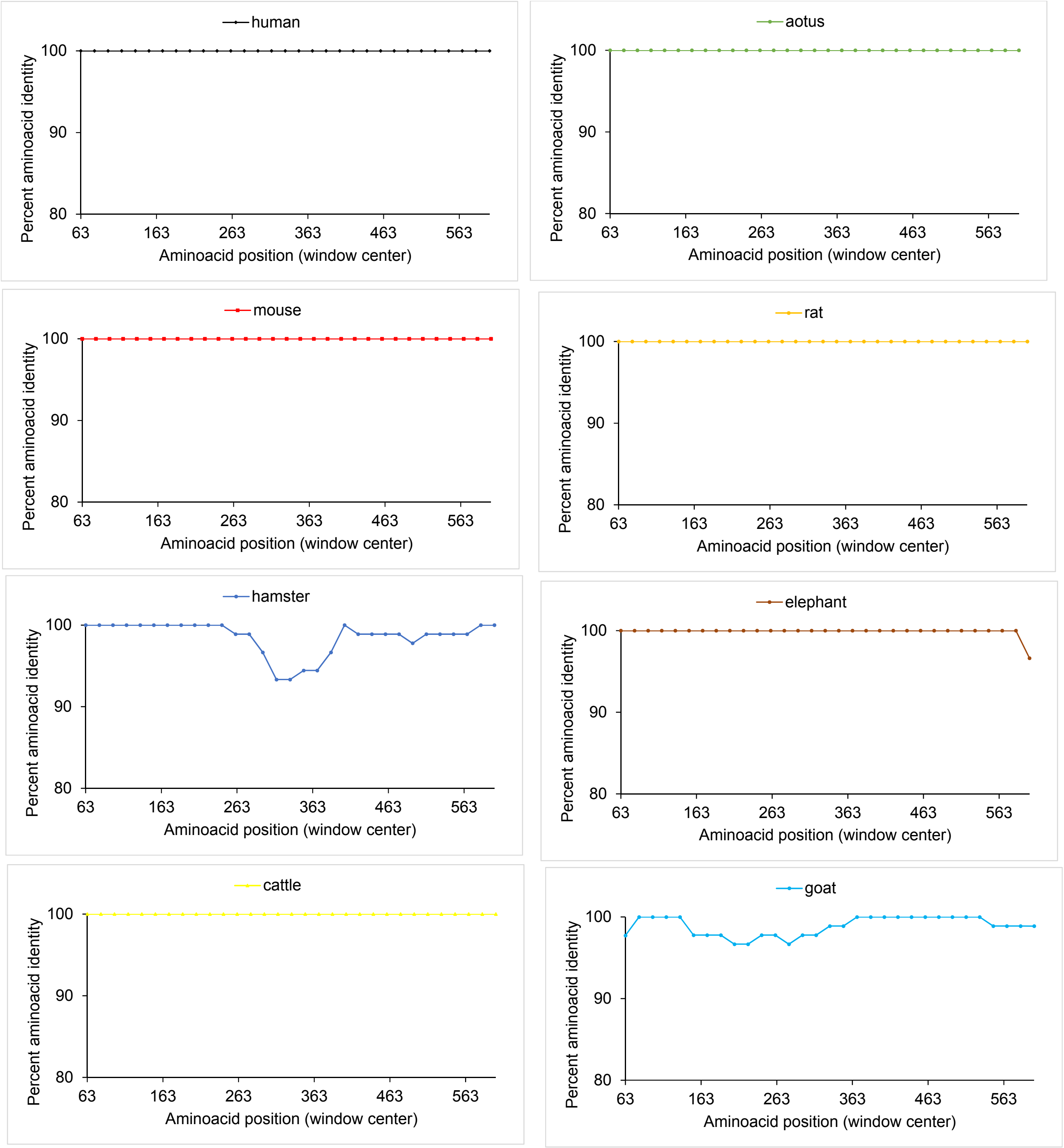

Supplementary Fig. S2

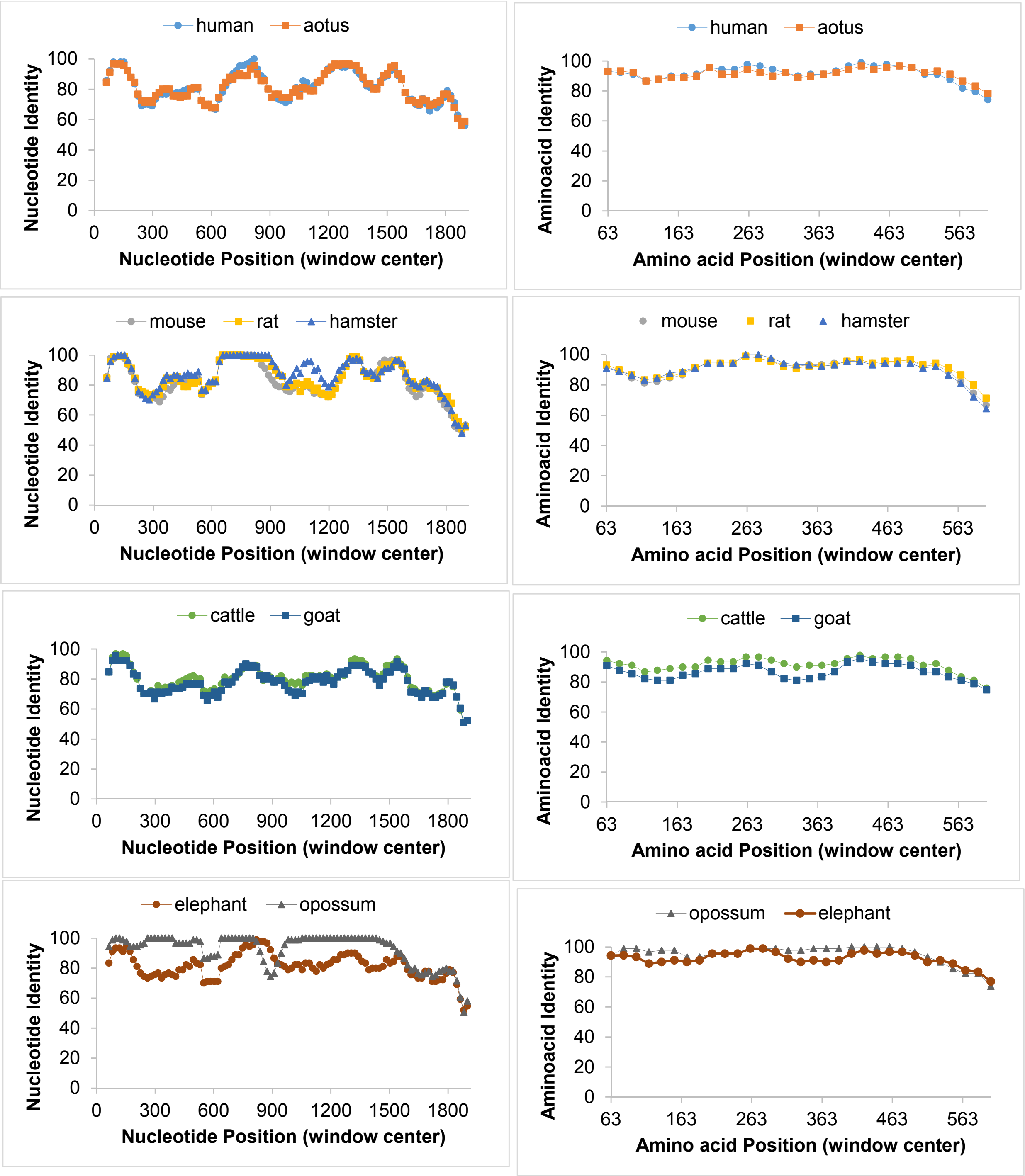

Supplementary Fig. S3

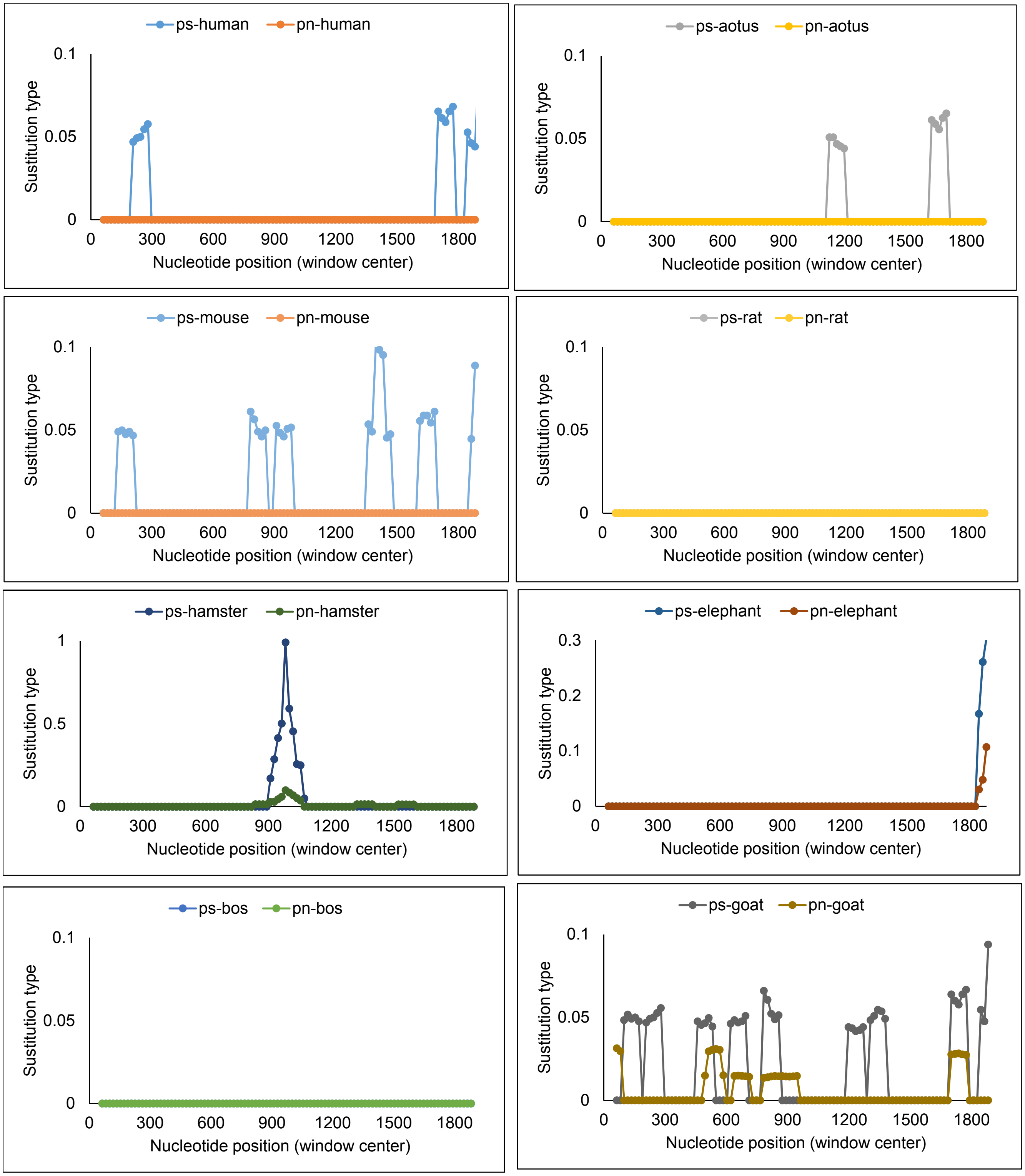

Supplementary Fig. S4 (A1A-A1B)

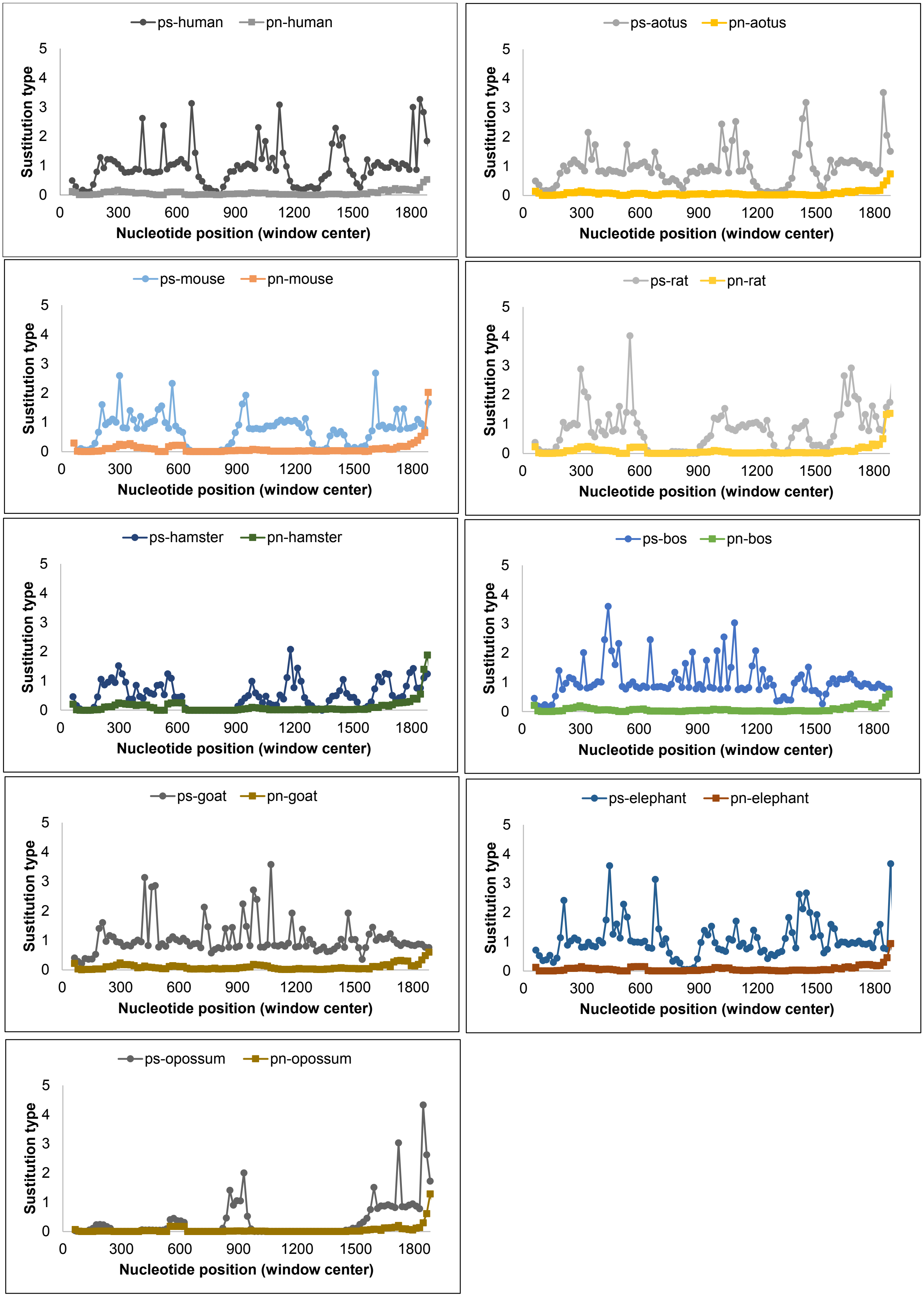

Supplementary Fig. S4 (A1A-A1L)

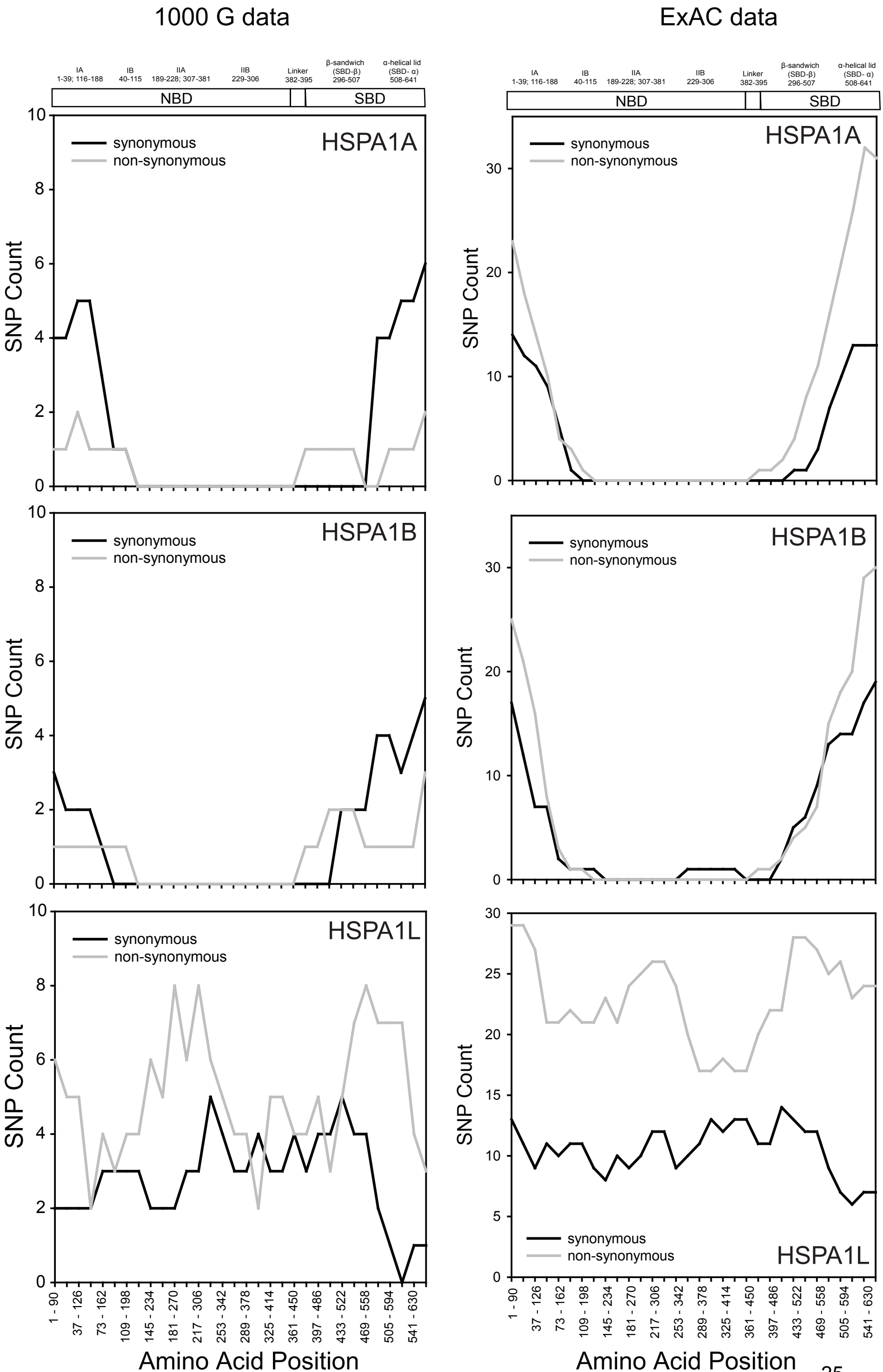

Supplementary Fig. S5

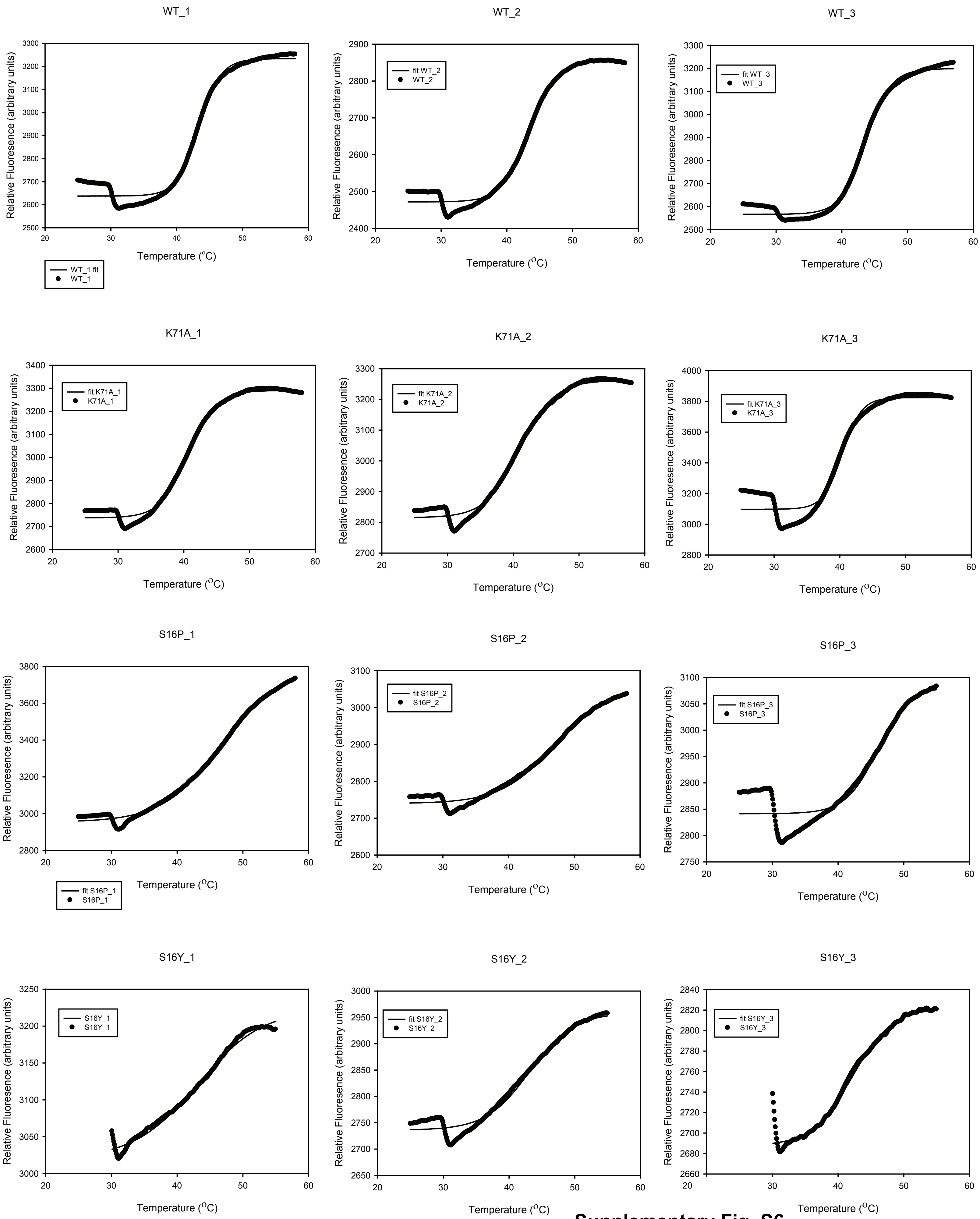

Supplementary Fig. S6

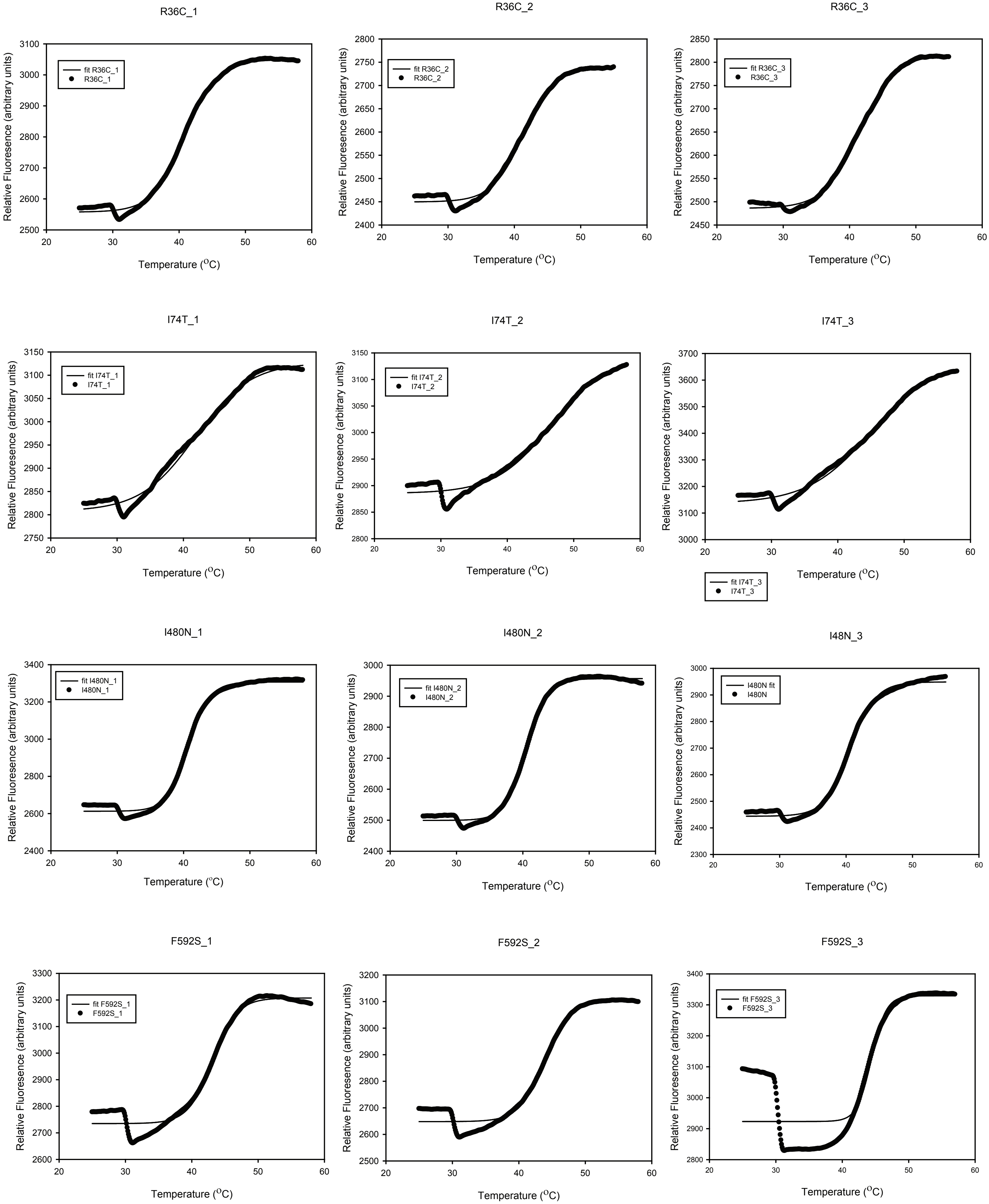

Supplementary Fig. S6

ATP ADP

Supplementary Fig. S7

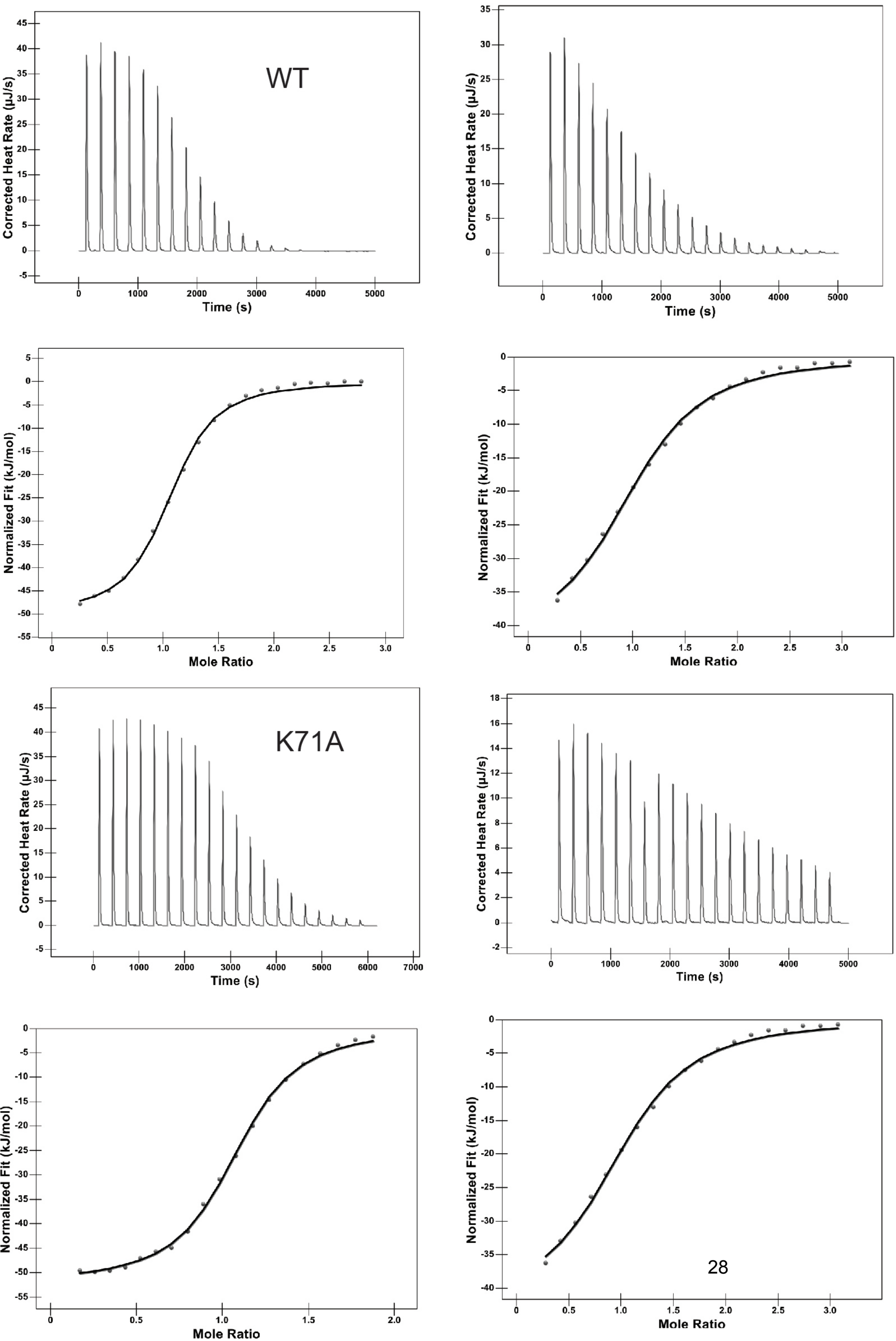

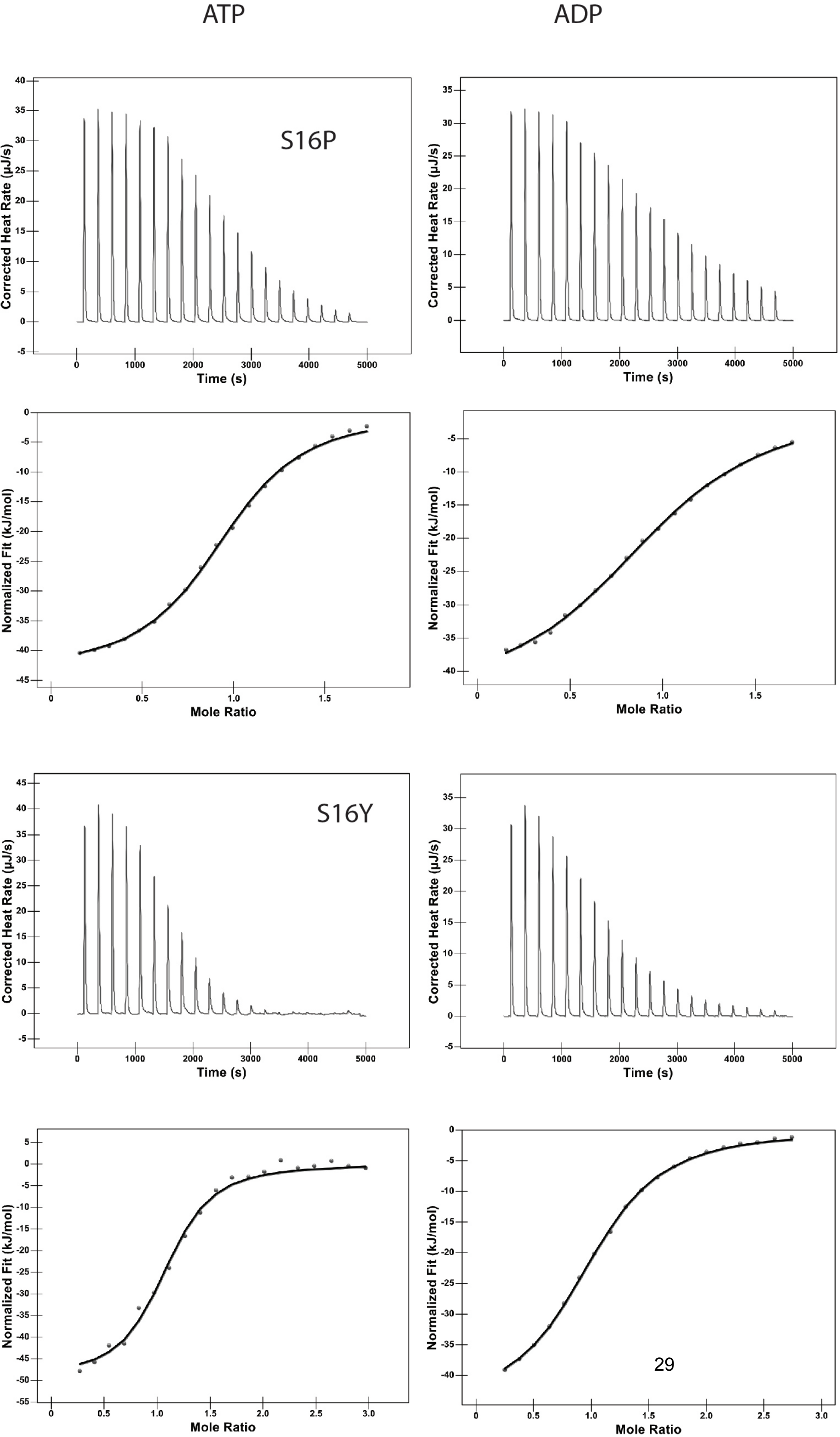
ATP
ADP
S16P
S16Y
Corrected Heat Rate (µJ/s)
Time (s)
Normalized Fit (kJ/mol)
Mole Ratio

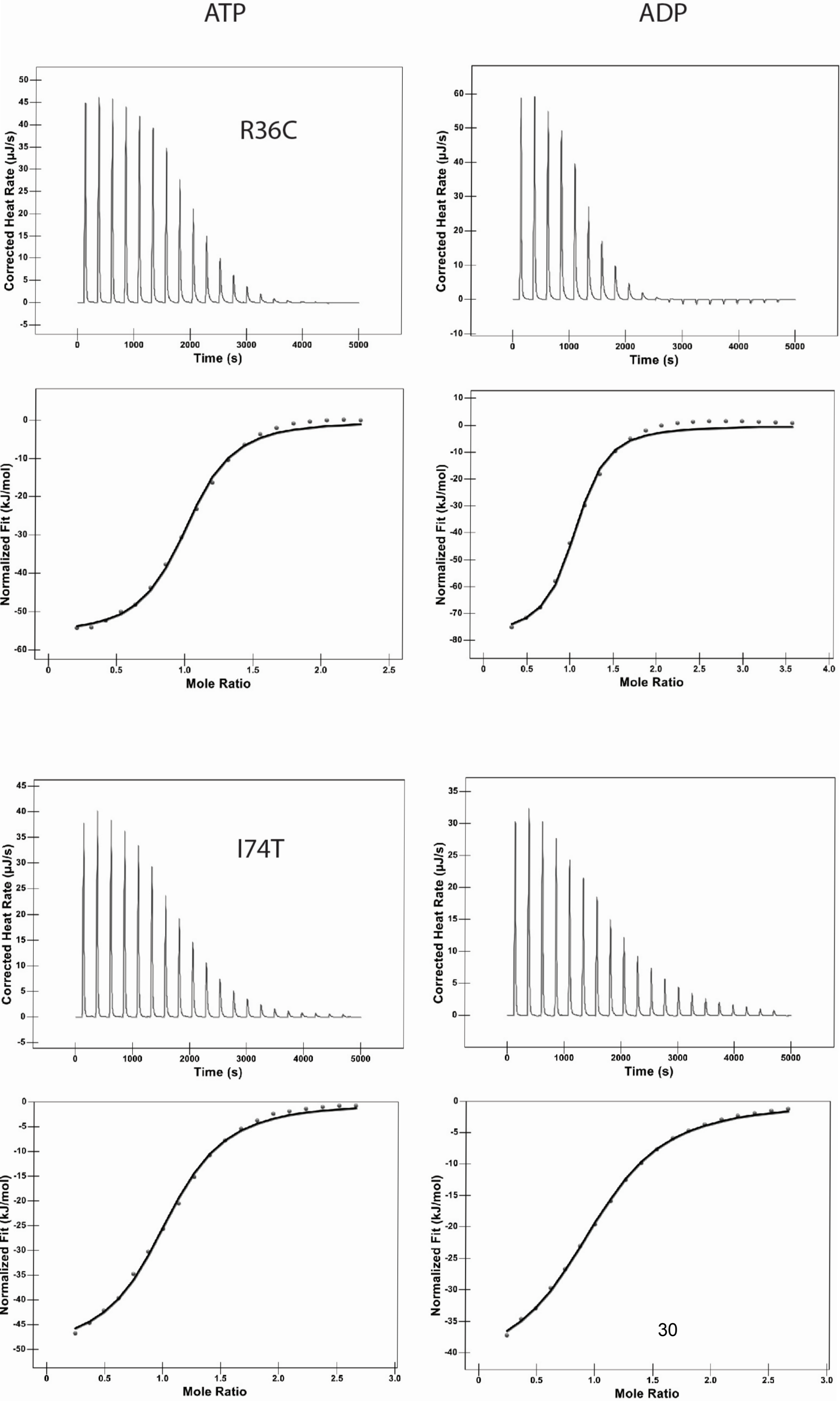
ATP
ADP
R36C
I74T
Corrected Heat Rate (µJ/s)
Time (s)
Normalized Fit (kJ/mol)
Mole Ratio

ATP ADP

Suppl. Fig. 7

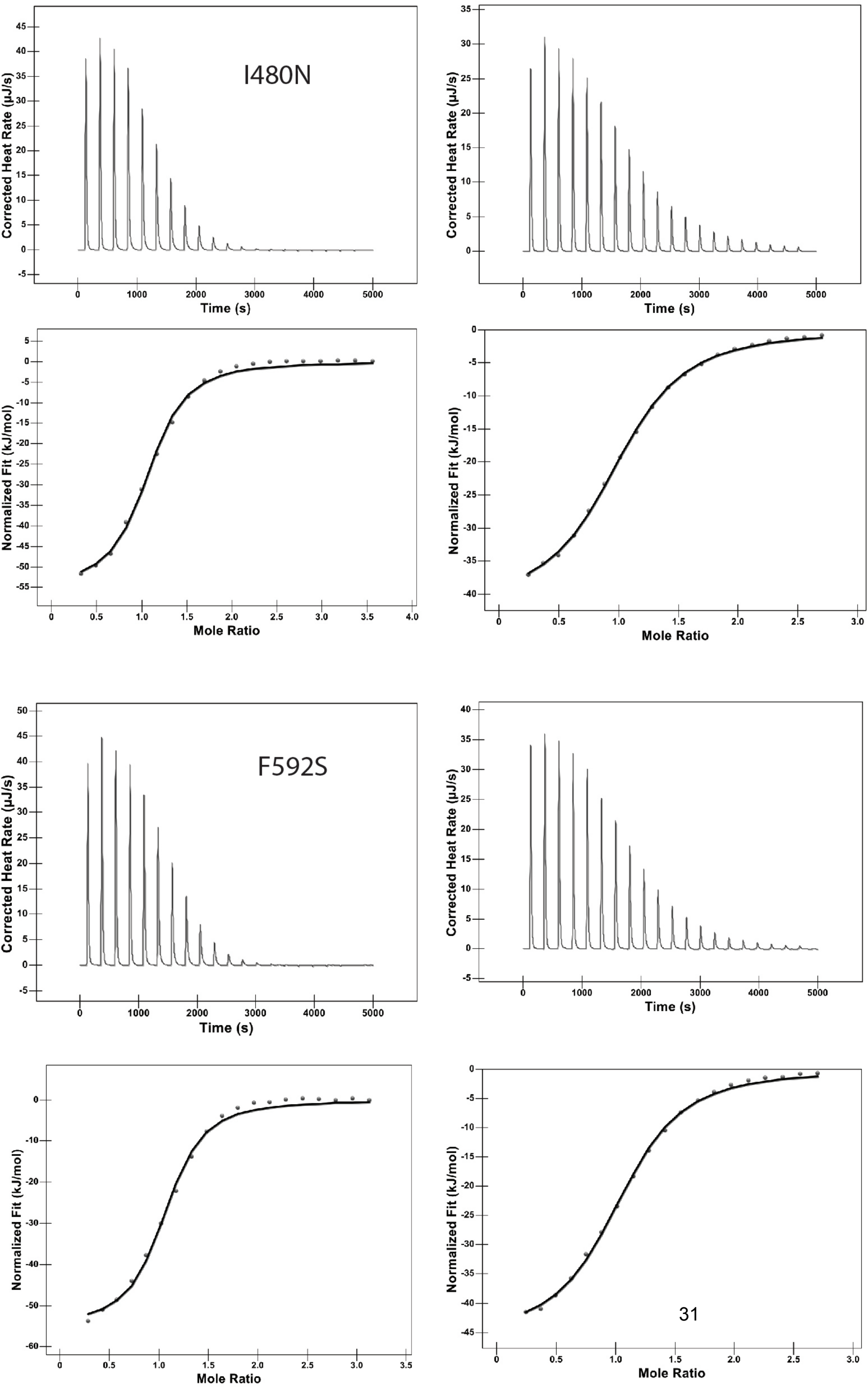

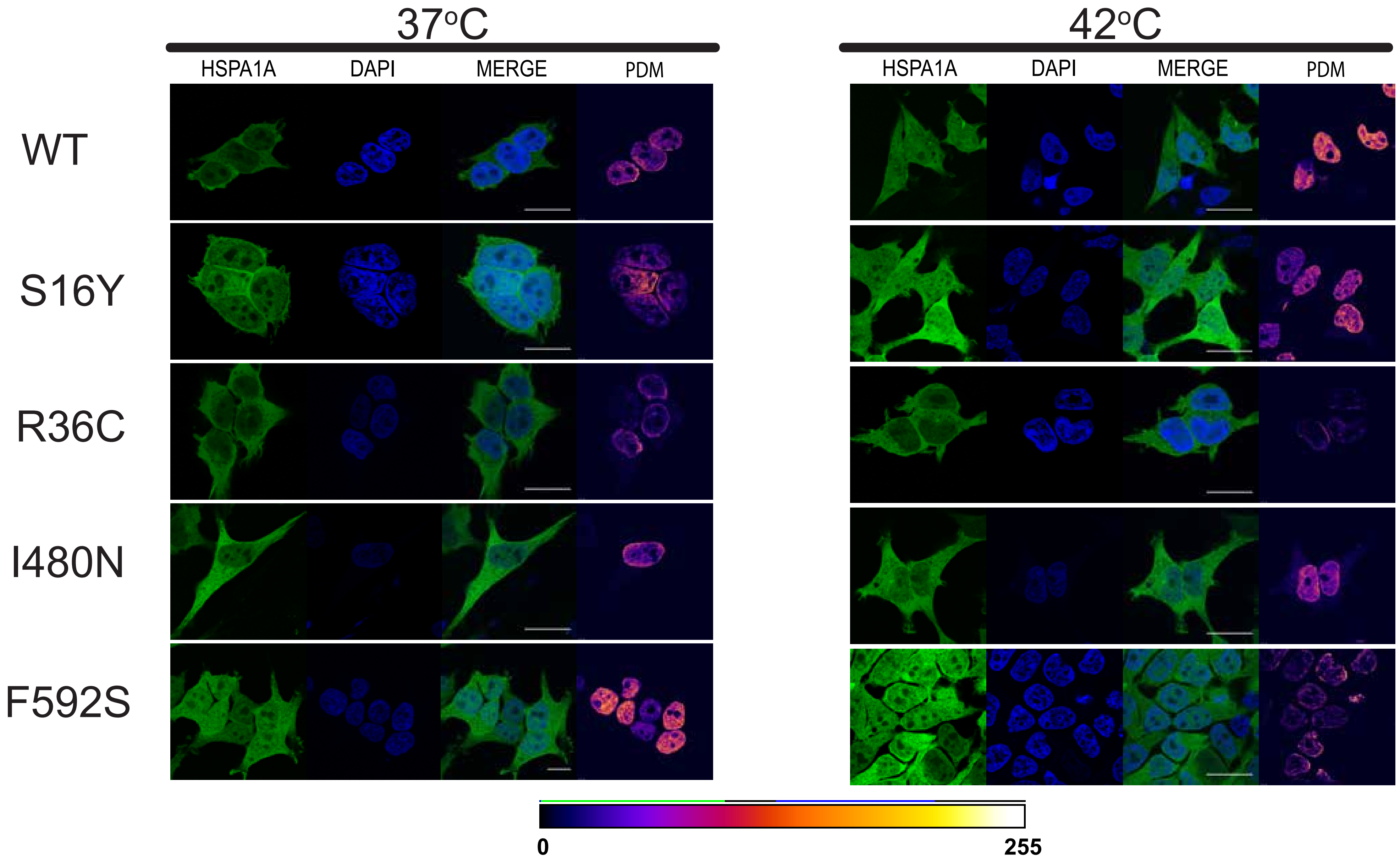

Supplementary Fig. S8

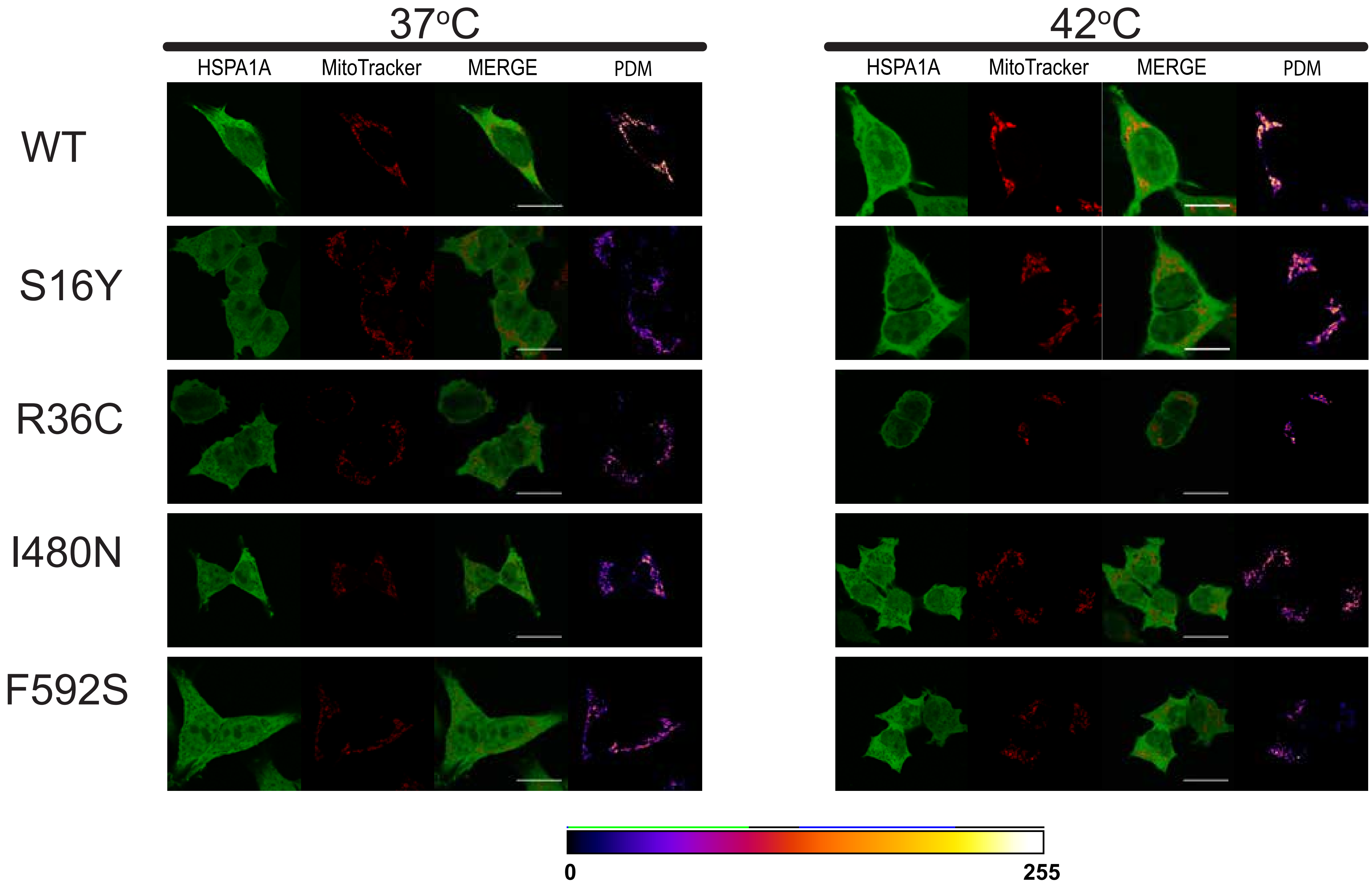

Supplementary Fig. S8

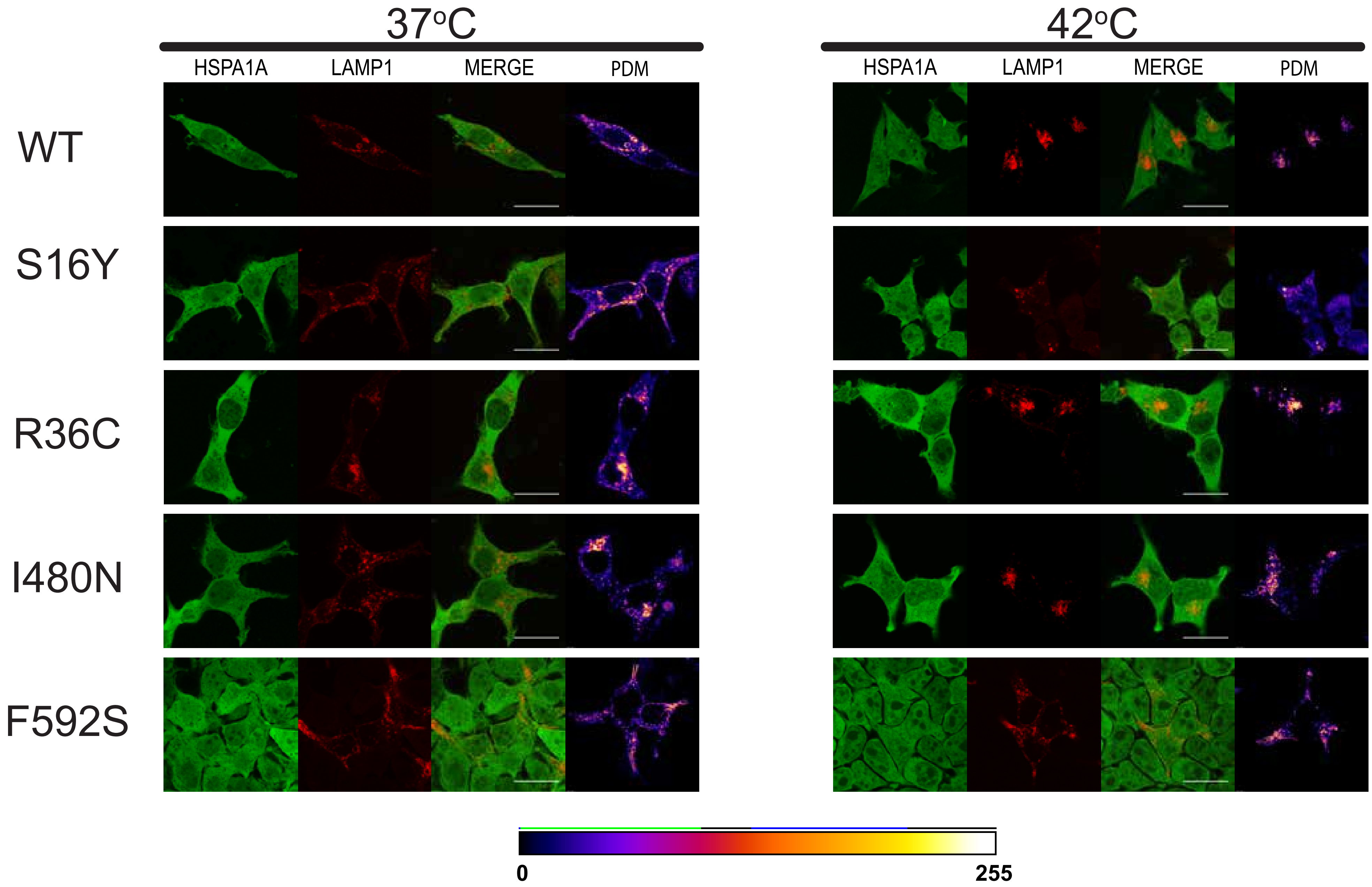

Supplementary Fig. S8

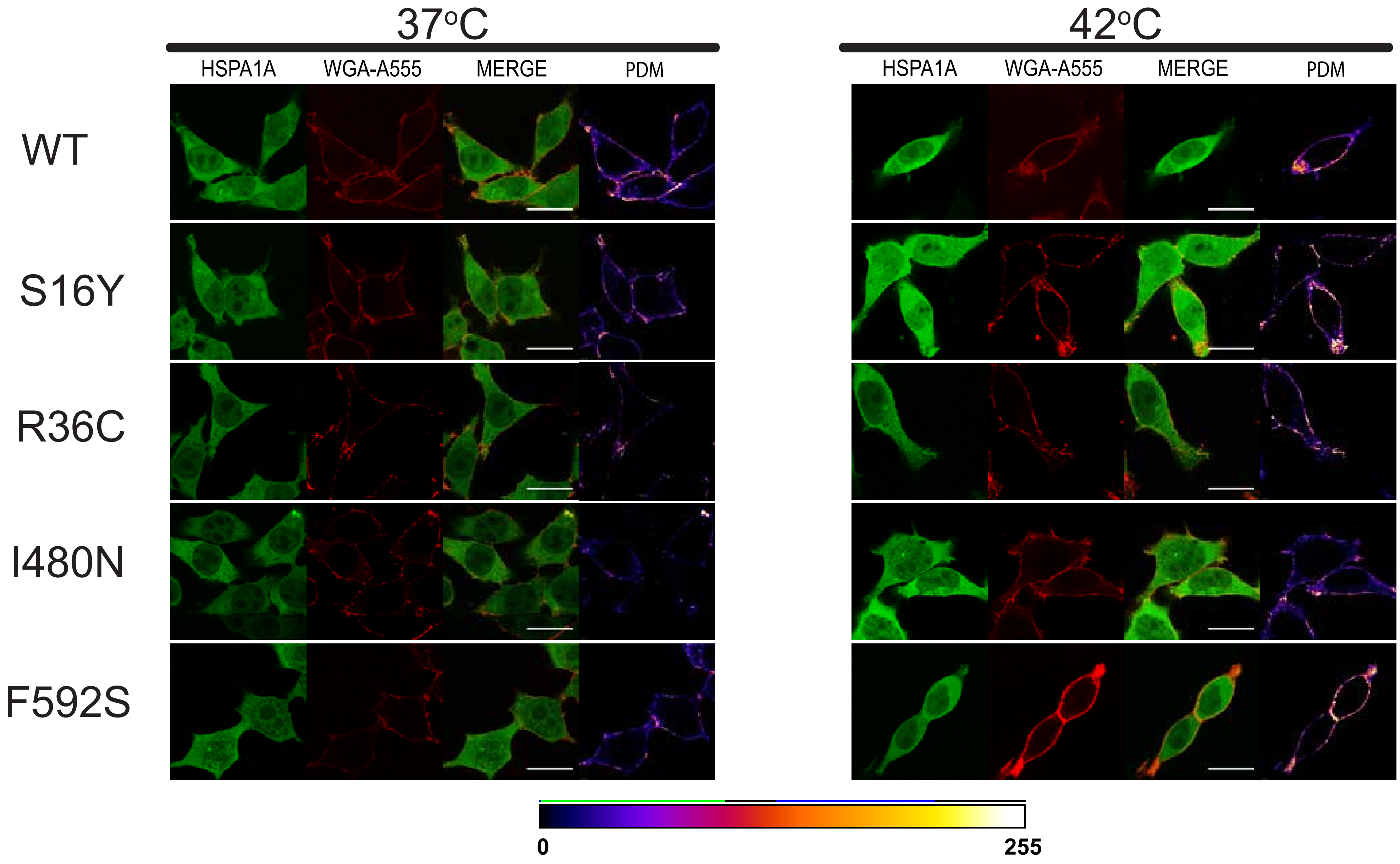

Supplementary Fig. S8
